# The Status of Honey Bee Health in Italy: Results from the Nationwide Bee Monitoring Network

**DOI:** 10.1371/journal.pone.0155411

**Published:** 2016-05-16

**Authors:** Claudio Porrini, Franco Mutinelli, Laura Bortolotti, Anna Granato, Lynn Laurenson, Katherine Roberts, Albino Gallina, Nicholas Silvester, Piotr Medrzycki, Teresa Renzi, Fabio Sgolastra, Marco Lodesani

**Affiliations:** 1 Dipartimento di Scienze Agrarie (DipSA), Università di Bologna, Bologna, Italy; 2 Istituto Zooprofilattico Sperimentale delle Venezie, NRL for beekeeping, Legnaro (Padova), Italy; 3 CRA-API, Consiglio per la Ricerca e la Sperimentazione in Agricoltura, Bologna, Italy; 4 FERA, Sand Hutton, York, United Kingdom; University of California San Diego, UNITED STATES

## Abstract

In Italy a nation-wide monitoring network was established in 2009 in response to significant honey bee colony mortality reported during 2008. The network comprised of approximately 100 apiaries located across Italy. Colonies were sampled four times per year, in order to assess the health status and to collect samples for pathogen, chemical and pollen analyses. The prevalence of *Nosema ceranae* ranged, on average, from 47–69% in 2009 and from 30–60% in 2010, with strong seasonal variation. Virus prevalence was higher in 2010 than in 2009. The most widespread viruses were BQCV, DWV and SBV. The most frequent pesticides in all hive contents were organophosphates and pyrethroids such as coumaphos and tau-fluvalinate. Beeswax was the most frequently contaminated hive product, with 40% of samples positive and 13% having multiple residues, while 27% of bee-bread and 12% of honey bee samples were contaminated. Colony losses in 2009/10 were on average 19%, with no major differences between regions of Italy. In 2009, the presence of DWV in autumn was positively correlated with colony losses. Similarly, hive mortality was higher in BQCV infected colonies in the first and second visits of the year. In 2010, colony losses were significantly related to the presence of pesticides in honey bees during the second sampling period. Honey bee exposure to poisons in spring could have a negative impact at the colony level, contributing to increase colony mortality during the beekeeping season. In both 2009 and 2010, colony mortality rates were positively related to the percentage of agricultural land surrounding apiaries, supporting the importance of land use for honey bee health.

## Introduction

In the last decade many concerns about the decline of honey bee (*Apis mellifera*) populations have been raised worldwide, with the potential for significant consequences to pollination services and honey production. Despite a global increase in the population of domesticated bees according to the FAO data [1], it is clear that honey bees have been facing growing adversity and that local declining events occurred in many European countries as well in the USA [2, 3, 4].

This phenomenon of losses is complex and may be driven by a number of factors including: colony collapse disorder (CCD) first described in US [5, 6], the depopulation of bee hives as observed in Europe, as well as declining honey bee numbers globally [7–10]. Spontaneous reports from beekeepers in Italy suggest that colony losses are likely to occur in two main periods across the year: a) during winter and at the beginning of the active season, when sudden honey bee decreases in population are recorded without any intoxication or pathogen symptoms; b) during spring time, when significant honey bee mortality coincides with field pesticide treatments.

Currently, the hypothesis of a multi-causal etiology of this phenomenon is well-accepted [11]: the synergistic effect of pesticides, infectious diseases, *Varroa* mite infestation, climate changes, poor nutritional sources and beekeeping practices may result in a substantial effect. In particular, the relationship between pesticide exposure and pathogen load has been investigated, unveiling synergistic effects as well as increased vulnerability mechanisms [12–17].

Several previous studies have investigated the in-hive pesticide contamination [18–24] and/or the presence of various pathogens [24–30] and have provided evidence to support the hypothesis of multiple causes of bee declines. Among the investigated factors the nutritional value of collected pollen is increasingly understood [31]. It has been demonstrated that a poor pollen diet can result in weak colony development and poor survival, due to the loss of immunocompetence [32] and the higher susceptibility to pesticides [33]. Therefore gathering and improving information about honey bee health status at local levels appears to be critical in order to better understand the causes of the phenomenon of honey bee decline. This has resulted in the setting up of a number of different networks across many European and Northern American countries [20, 21, 25]. In the recent EU regulation, it encourages member States to initiate monitoring programmes to verify the real exposure of bees to pesticides and chemicals [34].

In Italy significant spring mortalities of the honey bee have been recorded since 2003, and mainly correlated to the side-effects of maize seed dressing with neonicotinoid insecticides [35]. These reports markedly increased in 2008, leading to the creation of a National monitoring network providing regional monitoring activities [8, 36, 37]. The Italian Ministry of Agriculture Food and Forestry funded a biennial project (2009–2010) called “ApeNet: monitoring and research in beekeeping”, to establish a monitoring system across the different regional territories of Italy [36]. A network of more than 1,000 beehives across all Italian regions was set up, with apiaries selected according to the geographic and environmental characteristics.

Here we present the data collected in 2009 and 2010 through the ApeNet network. In particular, the survey data on (i) pathogen load (ii) pollen sources (iii) pesticide residues contamination and (iv) colony mortality from 2009 to 2010 is provided. This information was integrated into a database alongside the environmental data and analysed to aid understanding of the impact of these factors on honey bee health in Italy. Since 2011, this monitoring network was replaced by a new network, named BeeNet, with an increased number of apiaries, progressing up to 303 apiaries and approximately 3,000 colonies, some of this data will be also presented in comparison to the findings of the ApeNet data

## Material and Methods

### Organization and sampling

**Fig 1:**
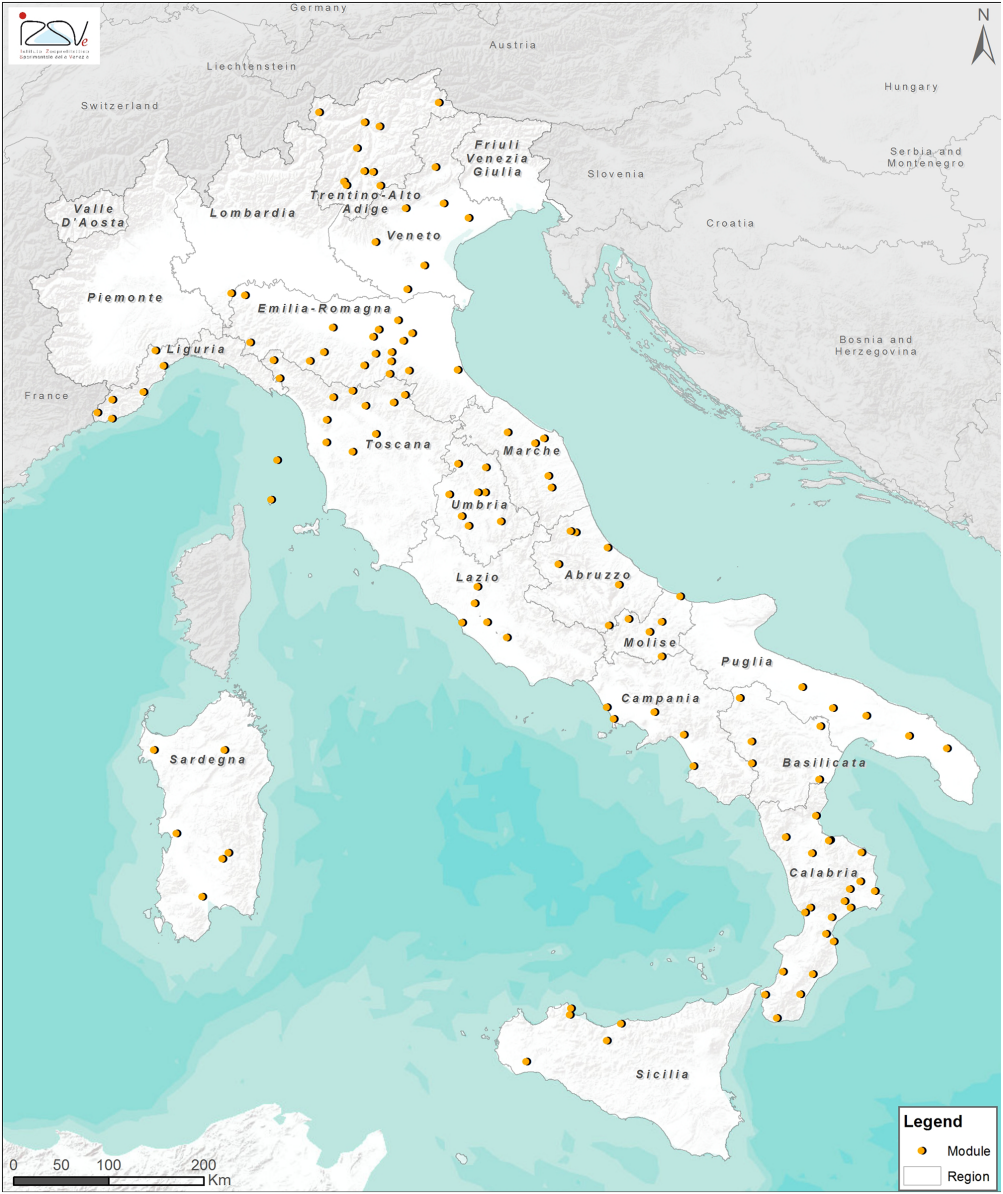
Location of the modules of the ApeNet monitoring network.

The Italian monitoring network consisted of 130 apiaries with 10 beehives in each (1,300 colonies), these were organized in 26 modules distributed across almost all of Italy’s geographic regions. These geographic regions were from three macro areas: North, Central and South of Italy (Fig 1, S1 Table). Each module consisted of five non-migratory apiaries. Beekeepers were enrolled in the monitoring network by the BeeNet project’s national management board and freely invited to participate. No specific permissions were required for these activities except for the explicit acceptance of the beekeepers. The field studies did not involve endangered or protected species.

Each colony was sampled four times a year (the end of winter, spring-summer, end of the summer-beginning of autumn; before over wintering). Each apiary was georeferenced, the land use (agricultural, urban, industrial or natural) was visually assessed at the beginning of the survey and updated in the following inspections, considering 1.5 km radius area surrounding the apiary. At each sampling point the colony status (number of bees, amount of brood, any symptoms of diseases, presence and age of the queen), nutritional status (abundance of pollen and honey) and behavioural traits (flight activity, aggressiveness, abnormal behaviours) were recorded. Samples of materials from within the beehives were also collected; 50 live forager honey bees per hive, 5x5 cm-wax cap per hive and 1.5 mL tube of bee-bread per apiary. This allowed for each apiary to have chemical (pesticides), pathological (*Nosema* spp, viruses) and nutritional (bee-bread raw proteins) analyses carried out four times a year. The samples were collected from each hive and then pooled for each apiary, therefore resulting in approximately 390 samples per sample time point, and 1500 samples per year, inclusive of the different hive materials (bees, bee-bread and wax). We considered the apiary as the epidemiological unit for our population study.

For parasite prevalence the live bee samples were stored at -20°C and at -80°C (in field, bees were collected in small cages and maintained alive until temporarily storage at -20°C and sent to the laboratory in dry ice in two weeks), respectively to be screened for *Nosema ceranae*, *Nosema apis* and eight commonly occurring viruses (Table 1). *Varroa* infestation has been assessed only with a symptomatic evaluation because at the time of this project a fast and practical field method was not available, like the powdered sugar test. The sample of bee-bread collected in each apiary was used to measure the level of raw proteins as an estimation of the nutritional quality of the stored food, while wax, bee-bread and live bees were also used for pesticide residue screening. Any colony losses during the year (including over-winter mortality) were recorded and, if a colony collapsed during the study, it was replaced with another colony from the same apiary in early spring of the next year. In case of swarming and queen loss, this information was recorded, and in the event of robbing, the hives were excluded from the count of lost colonies. All the collected data were registered in a database specifically created for monitoring data management called ApeNet. The ApeNet website was made available publicly via the internet and served a dual purpose of providing both a source of reference material and as a data entry/review system for all concerned with the project.

**Table 1:** List of analysed viruses and genome type.

VIRUS		GENOME	TAXONOMY
Deformed wing virus	DVW	Iflaviridae	ssRNA
Black queen cell virus	BQCV	Dicistroviridae	ssRNA
Sacbrood virus	SBV	Iflaviridae	ssRNA
Acute bee paralysis virus	ABPV	Dicistroviridae	ssRNA
Kashmir bee virus	KBV	Dicistroviridae	ssRNA
Chronic bee paralysis virus	CBPV	unclassified	ssRNA
Israeli acute paralysis virus	IAPV	Dicistroviridae	ssRNA
Apis iridescent virus	AIV	Iridoviridae	dsDNA

### Pathogen analyses

Crushed adult bees were examined on a microscope slide for detection of *Nosema* spp. spores by light microscopy at 400x [38]. The samples were also used for DNA extraction using the QIAamp DNA Mini Kit (Qiagen) according to the manufacturer’s instructions, with a lysozyme pre-incubation step. Negative controls (water for molecular biology applications in place of sample) were processed in parallel to detect possible contamination. The extracted DNA yield and purity (260/280 and 260/230 nm absorbance ratios) were checked by using a Nanodrop N1000 spectrophotometer (NanoDrop Technologies Inc.). DNA was stored at -20°C prior to use.

DNA was amplified using two different sets of primers published by Higes et al. [39] (NOS-FOR and NOS-REV) and Webster et al. [40] (NosA-F and NosA-R), respectively. PCR was performed in a total volume of 50 μL containing a final concentration of 1X PCR Buffer, 2.5 mM MgCl_2_, 0.2 mM dNTPS, 0.5 μM each primer (NOS or NosA), 1.25 U AmpliTaq Gold (Applied Biosystem, Foster City, CA, USA) and 100–200 ng of DNA. PCR was carried out using a GeneAmp^®^ PCR System 9700 (Applied Biosystem). PCR products were electrophoresed on 7% acrylamide gel, visualized by silver staining and compared to a commercial 100 bp size ladder. Positive (reference *N*. *apis* and *N*. *ceranae* DNA extracts as template) and negative (water for molecular biology applications instead of DNA template) controls were included in every PCR.

All PCR products were sequenced in both directions using the Big Dye Terminator v3.1 cycle sequencing kit (Applied Biosystem). The products of the sequencing reactions were purified using Performa DTR Ultra 96-Well kit (Edge BioSystems) and analysed in a 16-capillary ABI PRISM 3130xl Genetic Analyzer (Applied Biosystem). Sequence data were assembled and edited with SeqScape software v2.5 (Applied Biosystem) and the resulting sequences compared with the sequences of *N*. *apis* and *N*. *ceranae* available in GenBank using the BLASTN search.

For the viral screening Total Nucleic Acid (TNA) was extracted from 60 bees homogenized for 12 minutes with 20 mL GITC Lysis Buffer (5 M Guandine Thiocynate, 0.05 M Tris base, 0.02 M EDTA, pH 8.0) in a 30 mL bottle containing 3, 7/16” ball bearings. GITC Lysis buffer also contained 17.3 mM SDS buffer (173 mM Sodium dodecyl sulphate (SDS) in 100 mL MGW). The homogenate was then spun at 6189 g for 5 minutes. Polypropylene 96 -deep well plates (DT850301 Elkay Laboratory Products Ltd) were prepared as follows (1 well /extract); plate 1: 1000 μL extract, and 75μL MagneSil^™^ beads (MD1441, Promega); plate 2: 1 mL of GITC wash buffer (5M Guandine Thiocynate, 0.05M Tris base); plates 3, 4: 1 mL 70%v/v ethanol (E/00665DF/17, Fisher Scientific); plate 5: 200uL 1 X TE Buffer (stock 100X TE (EC-862 National Diagnostics). Virus primers used for DWV, KBV, IAPV, ABPV have been previously described in Martin et al. [41, 42, 43]. Novel primers for this testing are described in S2 Table. The 5’-terminal reporter dye for each *TaqMan*^®^ probe was 6-carboxyfluorescin (FAM) and the 3’ quencher was tetra-methylcarboxyrhodamine (TAMRA) or Minor groove binding (MGB) as indicated. Each sample was also tested for an internal control (Elongation Factor 1) [41].

Plates were loaded onto the Kingfisher 96 and processed as follows: Plate A—Bind 10 mins (fast dual mix), Plate B—Wash 3 mins (fast dual mix), Plate C,D—Wash 2 mins (fast dual mix), collect beads at 1 min intervals, Plate E—Mix 1 min then incubate at 65°C for 5 mins with mixing. TNA was collected from plate E of each reaction and stored at -80°C prior to use in real-time PCR.

Real-time PCR reactions were set up in 96 well reaction plates using TaqMan Gold core reagent kits (Applied Biosystems), following the supplied protocols. For the RNA viruses 1 unit of MMLV (EPO441: Fermentas) was added to each reaction. The primers (S2 Table) were all used at 300 nM and probes at 100 nM final concentration. TNA (1μL) was added to each reaction, giving a final reaction volume of 25 μL. A 7900HT Sequence Detection System (Applied Biosystems) was used for real time data collection. The results were recorded as the cycle threshold (Ct) or cycle number after which a significant accumulation of florescence over the baseline was observed; an average (of duplicate wells) Ct value below 40 was regarded as a positive result with a threshold ΔRn setting of 0.2.

### Chemical Analyses—Residues in bees, beeswax and bee-bread

A multi-residue analysis of 128 compounds was performed using a QuEChERS method adapted to bee samples, followed by LC-MS and GC-ECD (S3 and S4 Tables). Detailed information about protocol, reagents and instruments is available in S1 Protocol.

### Chemical Analyses—Nitrogen content in bee-bread

The nitrogen content was determined by the Kjeldahl method and crude protein content was calculated as total N x 6.25. The ISO 937:1978 standard adjusted to 0.5 g of bee-bread, previously dried at 70°C overnight was used to determine the nitrogen content. Detailed information about reagents and instruments is available in S1 Protocol.

### Statistical Analyses

#### Spatial and temporal distribution of pathogens, pesticide residues and pollen quality

The values distribution and the median of data from pathological (*Nosema* spp. and viruses) and chemical analyses (pesticide residues and raw protein in the bee-bread) were calculated and graphically reported for each sampling period and year. Thematic maps were also built to show the spatial distribution (in each Italian region) of the percentages of positive pathogen and pesticide samples and the average percentage of raw proteins in bee-bread. Statistical analysis was not applied to KBV and IAPV because they were so rarely found: one sample positive to KBV in 2009 and 5 in 2010 respectively, and only three samples positive to IAPV in 2010.

For each year and sampling period, the relationship between the percentage of raw proteins (arcsine-transformed) and the percentage of agricultural land use surrounding the apiaries (arcsine-transformed) was analysed via simple linear regression.

For each pesticide found, the acute LD_50_ (48 hours) was retrieved from the PPDB database (http://sitem.herts.ac.uk/aeru/iupac/index.htm) and EPA Ecotox database (http://cfpub.epa.gov/ecotox/advanced_query.htm) and converted from μg/bee to ng/g in order to evaluate the risk posed to bees by the contamination of the analysed sampled [22].

#### Association between pathogen occurrences

For each sampling period in every year, the correlation between all potential pairings of pathogens were investigated with a phi coefficient for dichotomous nominal-scale data and its significance was tested with a contingency table [44]. The phi coefficient is a measure of association for two binary variables. Bonferroni correction was applied to account for multiple testing.

#### Relationship between colony losses and potential stressors

The average and standard deviation of colony losses for each Italian region was calculated. An overall linear correlation analysis was performed between the colony mortality rate (arcsine-transformed) and; the percentage of the land surface used for agriculture (arcsine-transformed), and the percentage of raw proteins in the bee-bread (separately for each sampling period). A Student t-test was used for each sampling period to verify if a difference in the mean colony mortality exists between parasite prevalence (pathogen-positive apiaries and pathogen-negative apiaries) and pesticide occurrence (pesticide-positive apiaries and pesticide-negative apiaries). Only pathogens found with a frequency of at least 10% of the total analysed samples were considered in the statistical test. These analyses were performed with a subset of the samples (84 in 2009 and 51 in 2010). These excluded colonies for which the beekeepers did not provide any data on colony mortality. The analyses were performed separately for each year considering the annual mortality rate in 2009 (from spring 2009 to early spring 2010) and the seasonal mortality rate in 2010 (from late spring to winter 2010).

Multivariate analysis could not be performed due to missing data, a common issue with epidemiological large field monitoring studies. The missing data were randomly spread across the data set. Data completion methods are not suitable for two reasons: i) the gaps are numerous and present in almost all cases (so the complete cases are very few); ii) different apiaries cannot be considered as replicates, making thus unsuitable for filling via donors or by means.

For these reasons, in the present study the statistical analyses were based principally on each single factor.

## Results

### *Nosema* spp. prevalence

**Fig 2:**
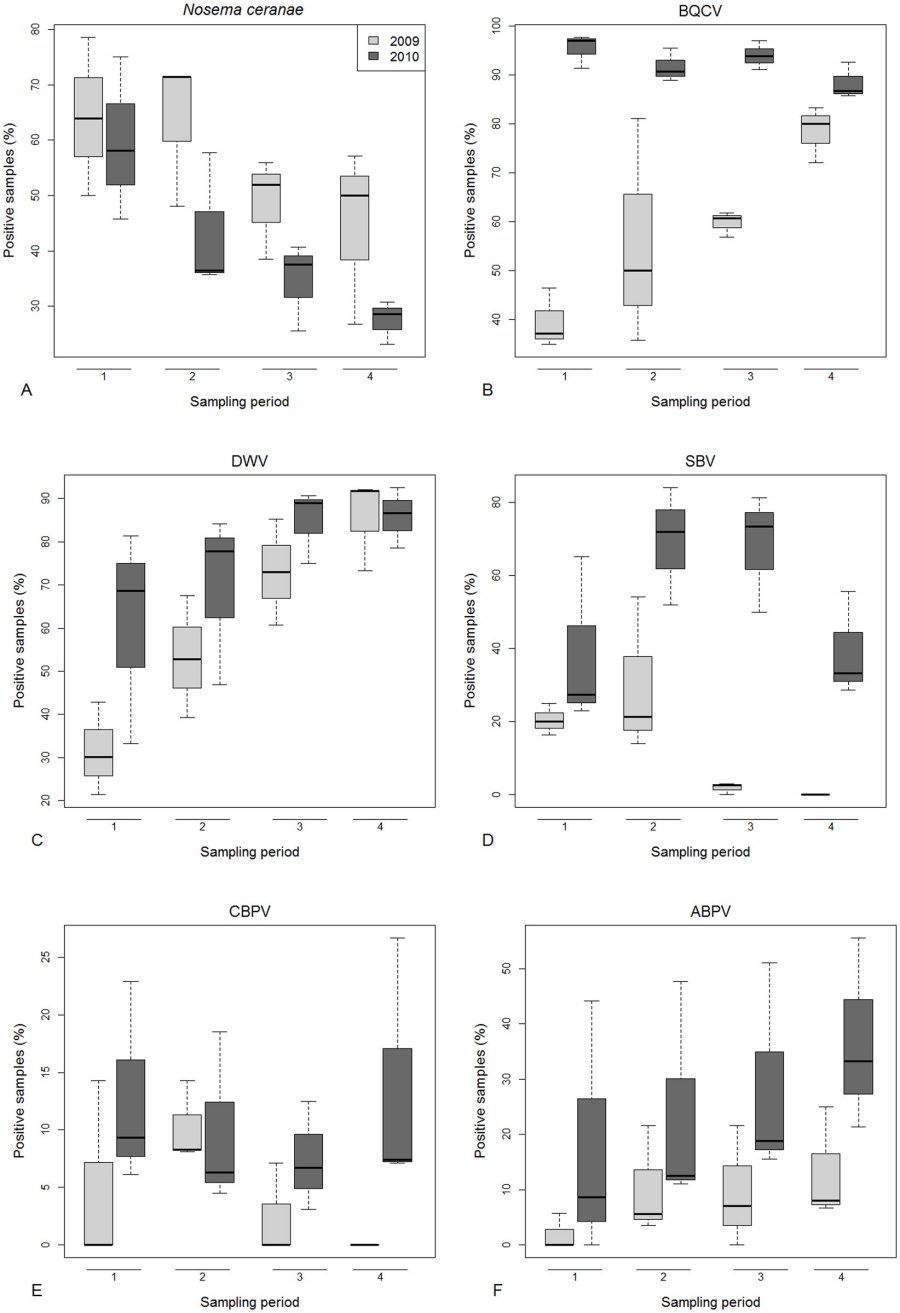
Pathogen infections: percentage over years and sampling periods of honey bee samples positive for *Nosema ceranae* (A), BQCV (B), DWV (C), SBV (D), CBPV (E) and ABPV (F). Boxes include 50% of the measured values and lines represent the median values. Whiskers include 90% of the data.

**Fig 3:**
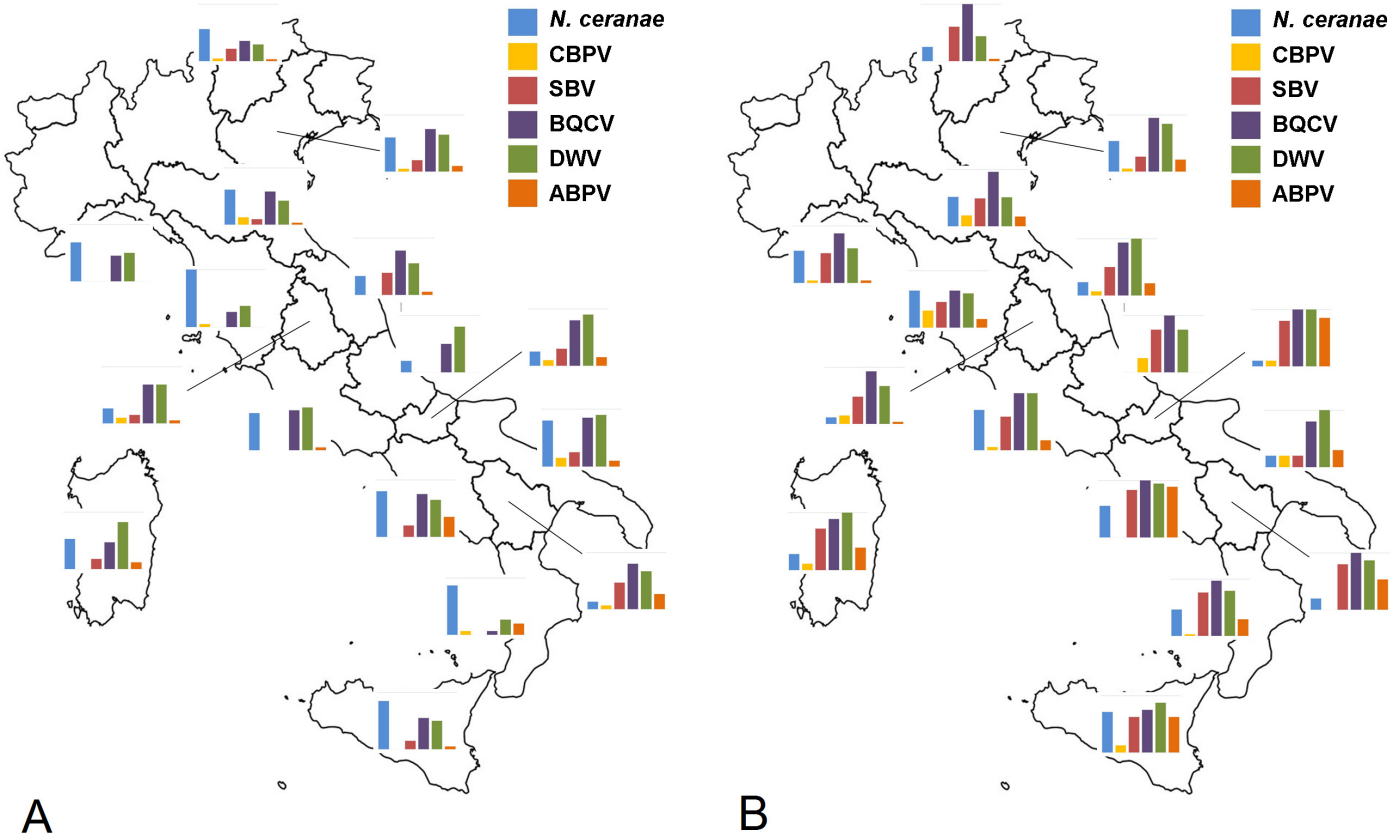
Pathogen occurrence: bars represent the percentage of positive samples of *Nosema ceranae*, CBPV, SBV, BCVV, DWV and ABPV, in 2009 (A) and 2010 (B). Values on y-axis range from 0 to 100%.

All the samples analysed during the monitoring period (2009 and 2010) were negative for *N*. *apis* however 49.5% of samples tested positive for *N*. *ceranae* (N = 649). A decrease in the percentage of positive samples was observed between 2009 and 2010. The level of *N*. *ceranae* infection was higher in the first sampling period and decreased during the year (Fig 2A). The distribution of *Nosema* spp. infection among Italian regions was not uniform (S5 Table, Fig 3).

### Virus prevalence

In both years of the study, the level of virus infection in Italy varied among periods and macro areas. Overall, the viruses found most frequently in adult honey bees (749 samples analysed) were BQCV (75%) and DWV (68%). SBV was found in approximately one third of the samples (33%), ABPV in 18% and CBPV in 8%, whereas KBV (0.8%) and IAPV (0.5%) were only rarely found. AIV was never detected.

For all viruses (BQCV, DWV, SBV, CBPV, ABPV) the percentage of positive samples was higher in 2010 than 2009, for DWV, SBV, CBPV and ABPV Southern Italian region had the highest prevalence of these viruses. For CBPV we found no clear spatial or temporal distribution trend (S5 Table, Figs 2 and 3). We found honey bees infected with KBV only in a few samples from Southern (2.9%) in 2009 and in samples from Northern Italy in 2010 (3^rd^ period: 12.5%; 4^th^ period: 6.7%). The IAPV was found only in 2010 and only in samples from Central and Southern Italy (S5 Table).

### Protein content in bee-bread

**Fig 4:**
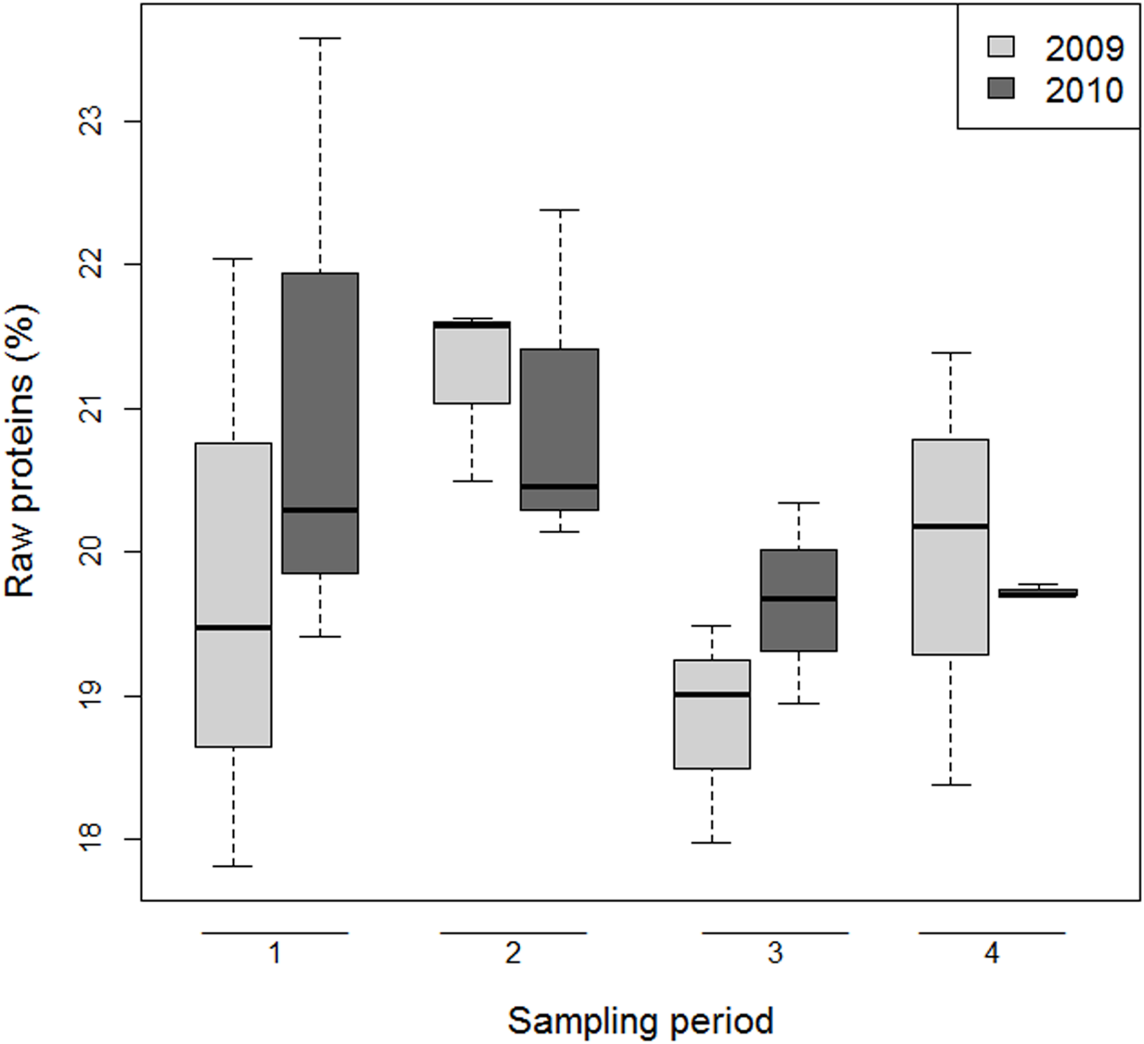
Pollen quality: percentage over years and sampling periods of raw protein in bee-bread. Boxes include 50% of the measured values and lines represent the median values. Whiskers include 90% of the data.

**Fig 5:**
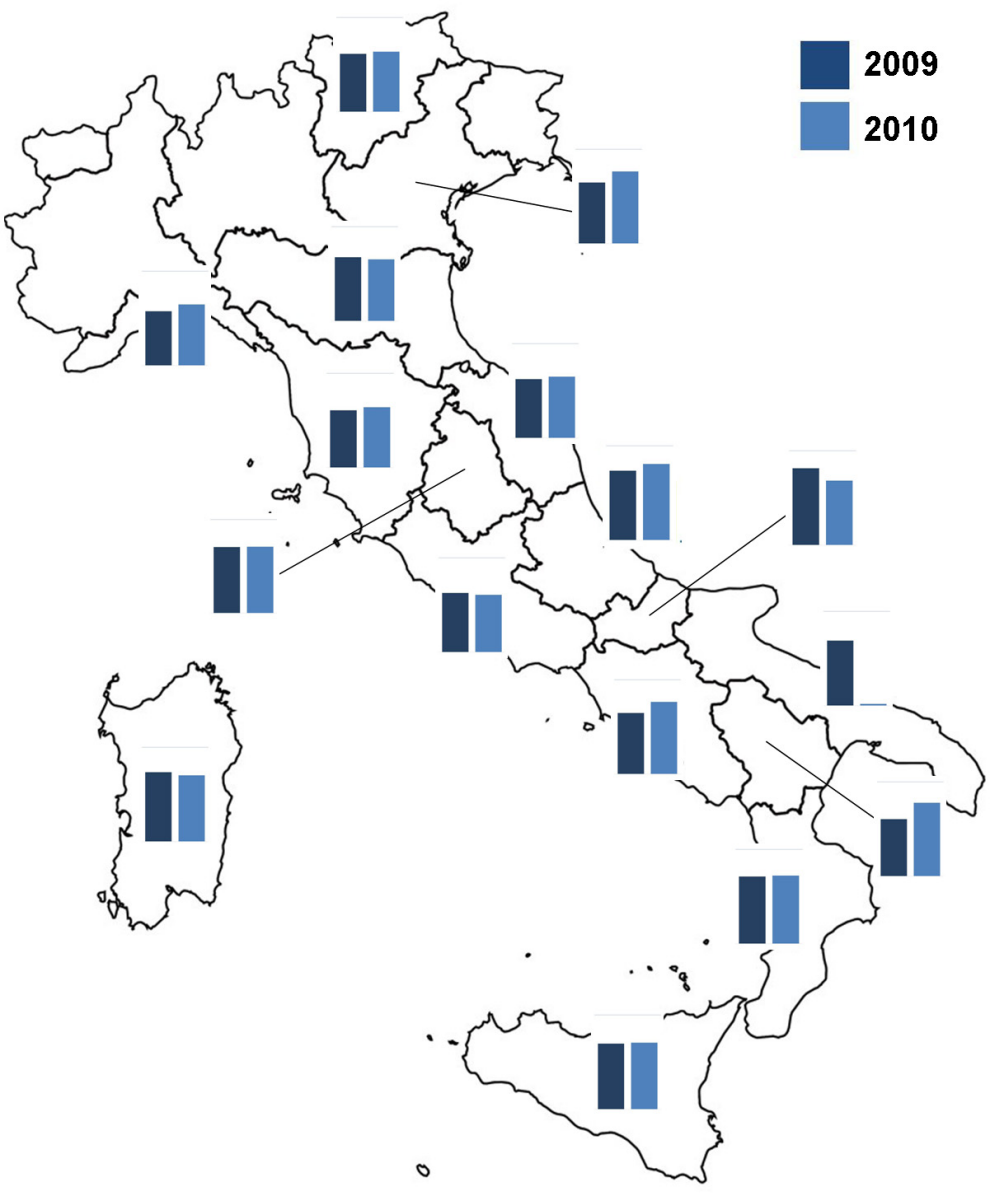
Pollen quality: bars represent the percentage of raw proteins in bee-bread in 2009 and 2010. Values on y-axis range from 0 to 30%.

The level of protein in bee-bread was similar between Southern (21.3%), Central (20.1%) and Northern Italy (19.2%). Overall, the percentage of raw protein in bee-bread was slightly higher in the first two periods (20.5%-21.1%) of the year than in the second semester (19.2%-20%). These results were similar in both years (S7 Table, Figs 4 and 5).

### Residues of pesticides in hive contents

**Fig 6:**
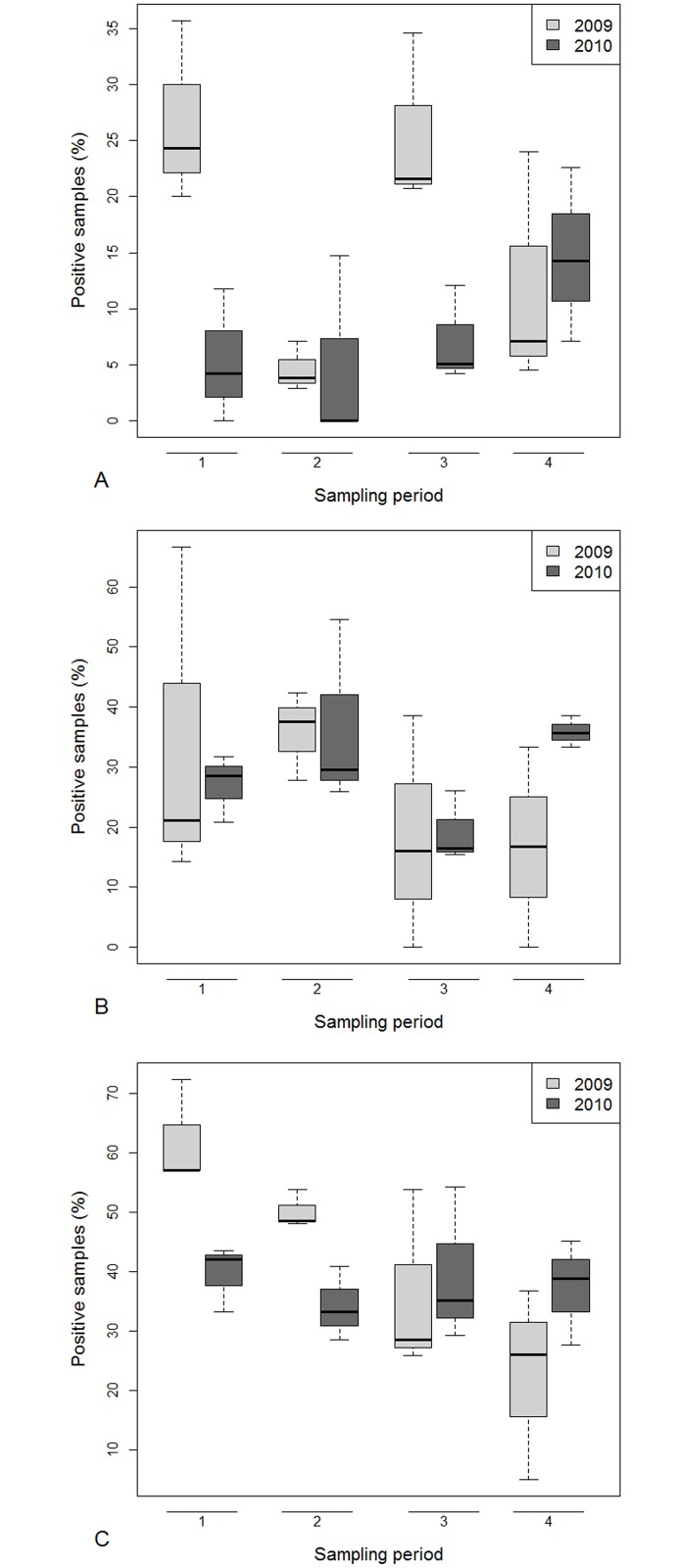
Pesticide contamination: percentage over years and sampling periods of honey bee (A), bee-bread (B) and beeswax (C) samples positive for pesticides. Boxes include 50% of the measured values and lines represent the median values. Whiskers include 90% of the data.

**Fig 7:**
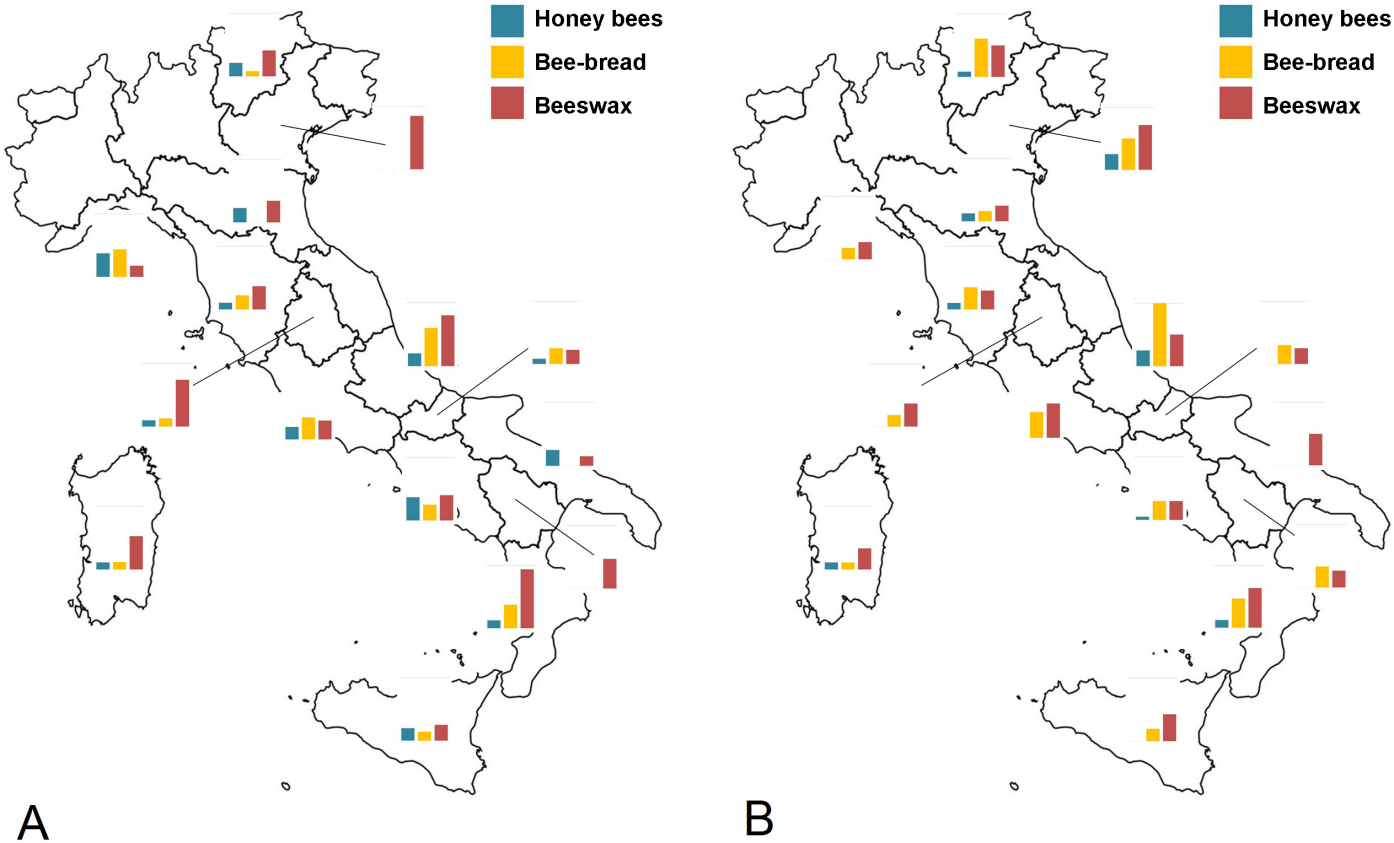
Pesticide contamination: bars represent the percentage of positive samples of honey bees, bee-bread and beeswax in 2009 (A) and 2010 (B). Values on y-axis range from 0 to 100%.

With a minimum detection threshold of 5 ng/g for every active ingredient studied in this work, the overall percentage of positive samples to pesticides was 12%, 27% and 40% respectively, for honey bees, bee-bread and beeswax. Samples with more than one residue were found in all types of matrices, particularly in beeswax, the most that was ever found in a matrix was four active ingredients. The percentage of honey bee samples positive for pesticides was higher in 2009 than in 2010, the first and third sampling periods were higher, with some differences among years and macro areas. In fact, the level of contamination decreased from 2009 to 2010 in all periods, except for the fourth sampling, particularly in Southern Italy (S8 Table, Figs 6 and 7). The percentage of bee-bread samples that were positive for pesticides was similar among years, macro areas and periods (S8 Table, Figs 6 and 7) and on average was 27.9% for beeswax, the percentage of samples positive for pesticides decreased linearly across the year and the sampling period, however, this trend was evident only in 2009. No clear spatial distribution was observed (S8 Table). Overall 34 different pesticides were detected with bee-bread being the matrix contaminated by the highest number of different pesticides (Table 2, S9 Table). In the majority of cases the active ingredients had a main use in agriculture, even though two are also in-hive compounds used against the *Varroa* mite (coumaphos, tau-flavalinate). In two cases residues of pesticides whose use is forbidden (chlorfenvinfos and rotenone) were found. The highest concentration residue found was coumaphos in beeswax (12779 ng/g), propamocarb in bee-bread (5,616 ng/g) and flumethrin in honey bees (452 ng/g). Compared with toxicity values, three neonicotinoids (imidacloprid, thiamethoxam, clothianidin) and fipronil were found in concentrations higher than LD_50_. In particular, clothianidin and imidacloprid were found in high concentrations in live bees, while thiamethoxam in bee-bread (Table 2). Insecticides were found mainly in honey bees and bee-bread samples, in particular organophosphates (chlorpyrifos ethyl and dimethoate), carbamates (pirimicarb) and neonicotinoids (clothianidin, imidacloprid, thiacloprid, thiamethoxam) (Table 2, S9 Table).

**Table 2:** Range of residue values (ng/g) in honey bees, beeswax and bee-bread samples from Italian bee colonies in 2009 and 2010.

#	Pesticide	Class*		2009			2010		
			LD_50_ (ng/g)	Honey bees	Beeswax	Bee-bread	Honey bees	Beeswax	Bee-bread
1	Acrinathrine	PYR	770	30	11–760	17–325	120	20–558	147–670
2	Benalaxyl	FUNG	>1000000	-	-	23	-	-	-
3	Bitertanol	FUNG	>1044000	10–119	12	-	-	-	-
4	Chlorfenvinphos	OP	5500	-	17–1157	22–215	15	27–2020	114–1560
5	Cyprodinil	FUNG	1125000	-	14–68	8–13	-	10–24	-
6	Chlorpyrifos ethil	OP	590	10–121	-	-	-	-	-
7	Clothianidin	NEO	40	36–103	-	-	15	-	12–99
8	Coumaphos	OP	75300	16–82	9–1428	68–1508	141–472	17–12779	22–446
9	Dimethoate	OP	1200	-	-	-	-	-	6–180
10	Dimethomorph	FUNG	>324000	82	-	-	-	-	245
11	Dithianon	FUNG	>254000	-	-	-	6–7	-	-
12	Fenamidone	FUNG	>1598000	-	-	-	-	-	212
13	Fenbuconazole	FUNG	>52000	-	-	-	125	-	-
14	Fenpyroximate	ACAR	>1185000	-	-	-	-	-	53
15	Fipronil	PHE	41,7	-	-	-	-	78	-
16	Fludioxonil	FUNG	<1000000	13–25	-	7–44	7–71	-	147–271
17	Flumethrin	PYR	500	452	12–89	23–34	18–42	12–145	58–464
18	Fluvalinate	PYR	35420	10–103	6–1346	30–1137	15–183	8–3000	16–1537
19	Imidacloprid	NEO	37	14–66	10–20	14	86	-	20–99
20	Kresomix methyl	FUNG	<1000000	22	-	446	-	-	167
21	Metalaxil	FUNG	2000000	-	40	10	-	10–36	10–20
22	Metamitron	HERB	>972000	-	11–23	9	-	-	-
23	Methomyl	CARB	1600	10–23	-	-	-	-	-
24	Oxamyl	CARB	3800	-	-	-	-	-	87–96
25	Piperonyl butoxide	SYN	2940000	-	7	-	-	-	-
26	Pirimicarb	CARB	40000	-	14–64	23	286	12–110	36
27	Propamocarb	FUNG	1000000	-	-	10–5616	11–46	41	52–2328
28	Pyrimethanil	FUNG	<1000000	-	-	62	-	10–53	18–584
29	Rotenone	NP	16660	11–140	53–709	37–380	-	-	23–60
30	Tebuconazole	FUNG	>830500	-	-	-	18–55	-	263
31	Teflubenzuron	IGR	720000	13	-	-	12	-	-
32	Thiacloprid	NEO	173200	11	-	-	-	-	-
33	Thiamethoxam	NEO	50	-	16	-	-	-	14–1619
34	Thiophanate methyl	FUNG	<1000000	-	-	-	-	-	302

* Class: ACAR = acaricide, CAR = carbamate, FUNG = fungicide, HERB = herbicide, IGR = Insect Growth Regulator, NEO = neonicotinoid, NP = natural product, OP = organophosphate, PHE = phenylpyrazole, PYR = pyrethroids, SYN = synergist

### Associations between pathogen occurrences

We found a positive association between the presence of ABPV and CBPV (phi = 0.49, p < 0.001) and between DWV and BQCV (phi = 0.37, p < 0.001) in the 1^st^ sampling period and six other positive associations in the 2^nd^ period of the 2009 data: ABPV/DWV (phi = 0.32, p = 0.02); ABPV/SBV (phi = 0.43, p < 0.001); ABPV/BQCV (phi = 0.30, p = 0.005); DWV/SBV (phi = 0.38, p < 0.001); DWV/BQCV (phi = 0.42, p < 0.001); SBV/BQCV (phi = 0.48, p < 0.001). No other significant association was observed in the 3^rd^ and 4^th^ period (S10 Table).

In 2010, ABPV was positively associated with DWV in the 1^st^ period (phi = 0.35, p = 0.001) and three other positive associations were observed in the 2^nd^ period: DWV/SBV (phi = 0.33, p = 0.003); DWV/BQCV (phi = 0.33, p = 0.003); SBV/BQCV (phi = 0.33, p = 0.003). We found only a significant negative association in the 3^rd^ period between CBPV and BQCV (phi = -0.41, p < 0.001) (S11 Table).

### Relationship between stressors and colony losses

In 2009 (from spring 2009 to early spring 2010) and in 2010 (from late spring to winter 2010), the colony mortality rate was, 19.17% (SD = ±20.13%, N = 84) and 7.23% (SD = ±19.97%; N = 97) respectively. The annual mortality rate in 2009 ranged from 4 to 34% among the Italian regions whereas, in 2010, the colony losses ranged from 0 to 20% (S12 Table).

The colony losses were positively related with the percentage of agricultural area surrounding the apiaries both in 2009 (r = 0.234; p = 0.032) and 2010 (r = 0.211; p = 0.038). In this study, the percentage of agricultural areas ranged from 0 to 100%, with an average of 54.5%. The altitude of the apiaries was negatively related to colony losses in 2009 (r = -0.321; p = 0.003) but not in 2010 (r = -0.094; p = 0.362). The colony mortality was also not significantly related to the raw protein content of bee-bread for each sampling period, both in 2009 and 2010 (Table 3).

**Table 3:** Linear correlation (r) between the colony mortality rate (%) and the percentage of land surface used for agriculture along with the percentage of raw proteins in bee-bread for each sampling period. p = p value.

Period	2009		2010	
	r	p	r	p
1°	-0.004	0.970	0.173	0.165
2°	0.078	0.514	-0.019	0.878
3°	0.082	0.512	-0.002	0.988
4°	-0.064	0.693	0.143	0.391

In 2009, the total colony losses was significantly higher in *Nosema*-positive colonies in the first sampling period, in DWV-positive colonies in the fourth sampling period and in BQCV-positive colonies in the first and second sampling periods (Table 4). No difference was detected in 2010 between colonies negative and positive for pathogens (Table 4). Among colonies positive for pesticides, higher colony losses were found when the pesticide residues were detected in adult bees, however, this difference was significant only in the second sampling period of 2010 (Table 5).

**Table 4:** Percentage of colony losses in apiaries non-infected (negative) and infected (positive) by different pathogens in each sampling period of 2009 and 2010. p value in italics indicates statistically significant difference (t-Student test, p <0.05). Only pathogens found in at least 10% of the analysed samples were considered in the test.

Colony losses (%)						
Pathogen / period	2009			2010		
	Negative	Positive	p	Negative	Positive	p
Nosema/1	12.8±3.49	23.65±2.88	*0*.*016*	5±1.75	7.9±1.85	0.247
Nosema/2	15.38±3.25	21.23±2.84	0.170	7.73±2.03	4.85±1.58	0.351
Nosema/3	17.63±3.53	22.93±2.88	0.150	9.11±1.98	3.87±1.1	0.173
Nosema/4	28.7±4.76	25.65±3.92	0.524	7.14±2.34	6±2.54	0.976
APBV/1	Infection < 10%			7.88±1.61	3.33±1.88	0.257
APBV/2	18.8±2.29	22.22±7.78	0.803	6.78±1.61	8.33±3.05	0.670
APBV/3	18.65±2.13	28.75±11.72	0.184	8.39±1.72	6.19±2.71	0.371
APBV/4	26.75±3.27	28.75±6.93	0.725	8.67±2.66	3.16±1.54	0.154
CPBV/1	Infection < 10%			5.67±1.13	16.36±6.78	0.058
CPBV/2	19.2±2.32	18.89±7.35	0.732	6.76±1.52	10±4.08	0.310
CPBV/4	Infection < 10%			6.9±2.03	4.29±2.02	0.954
DWV/1	17.37±2.68	26.5±4.43	0.062	7.74±1.78	6.81±2.02	0.340
DWV/2	18±3.13	20.23±3.11	0.822	7.62±2.17	6.96±1.78	0.492
DWV/3	16.36±3.7	20.83±2.72	0.375	11.82±4.23	7.12±1.54	0.217
DWV/4	15±7.19	28.81±3.13	*0*.*038*	4±4	6.82±1.92	0.619
SBV/1	19.17±2.32	21.76±6.76	0.911	6.88±1.69	7.67±2.48	0.880
SBV/2	19.14±2.74	19.23±3.68	0.900	8.64±2.57	6.55±1.71	0.476
SBV/3	Infection < 10%			7.78±2.47	7.8±1.81	0.950
SBV/4	Infection < 10%			5.56±1.8	7.73±3.29	0.850
BQCV/1	14.57±2.3	27.42±4.35	*0*.*009*	4±2.45	7.4±1.49	0.736
BQCV/2	14.05±3.28	23.19±2.85	*0*.*022*	8.33±4.77	7.04±1.49	0.644
BQCV/3	19.69±3.09	19.6±3.09	0.911	2±2	8.19±1.54	0.292
BQCV/4	20±6.24	28.72±3.29	0.186	4.29±2.97	6.9±2	0.713

**Table 5:** Percentage of colony losses in apiaries contaminated (positive) or uncontaminated (negative) by pesticides considering hive materials and sampling periods. Data for 2009 and 2010. p value in italics indicates statistically significant difference (t-Student test, p <0.05).

% Colony losses						
Hive material / period	2009			2010		
	Negative	Positive	p	Negative	Positive	p
Bee/1	21.51±2.59	16.32±5.78	0.235	7.53±0.84	10±5	0.200
Bee/2	19.18±2.44	16.67±8.82	0.907	6.41±0.73	22±9.84	*0*.*009*
Bee/3	19.29±2.81	20.53±4.43	0.793	7.69±0.87	8±3.58	0.927
Bee/4	25±3.14	22.5±6.2	0.982	6.92±1.11	8±2.53	0.777
Wax/1	17.6±3.02	21.28±3.37	0.847	7.07±1.1	7.95±1.2	0.922
Wax/2	19.17±2.83	19.5±3.7	0.690	6.67±0.93	8.44±1.49	0.385
Wax/3	20±3.23	19.63±3.4	0.908	7.92±1.14	7.43±1.26	0.816
Wax/4	22.56±3.59	31.67±5.48	0.214	9.71±1.64	6.8±1.36	0.623
Pollen/1	20±3.76	14.55±2.82	0.889	6.88±0.99	7.39±1.54	0.801
Pollen/2	18.71±3.46	11.82±3.25	0.451	7.5±1.08	5.2±1.04	0.396
Pollen/3	21.03±2.91	15±4.42	0.191	8.43±1.18	6.32±1.45	0.866
Pollen/4	24.44±3.79	35±15	0.435	9.13±1.9	5.71±1.53	0.219

## Discussion

In this study we monitored the honey bee colonies in Italy during 2009 and 2010 with the aim to collect information on the overall health status of honey bees and to identify the main stressors involved in colony losses. Our results showed that no single risk factor was found to be a significant marker for the subsequent collapse of the monitored colonies, but highlighted numerous factors which contribute to the global health status of colonies. In particular, the presence of viruses including DWV and BQCV, together with the land use significantly influences overall mortality. However, we acknowledge some limitations in our study due to the lack of *Varroa* assessment and the quantification of virus load. This was due to the absence, when we started the survey, of a standard method to assess *Varroa* infestation in the field and the unavailability of a fast and cheap method to quantitatively analyse the pathogen infections (e.g. virus load). These shortcomings were overcome discussing the results of ApeNet network with the information gathered through the BeeNet monitoring network where the sampling and methodological scheme was the same but with the introduction of quantitative assessment for pathogens (virus and *Nosema* spp.) and *Varroa* mite. The BeeNet scheme analysed more than 1,400 samples between 2011 and 2014 (data not shown).

### Pathogen prevalence

The emergent pathogen *N*. *ceranae* (Microsporidia) is widespread in Europe [45], and its presence appears to dominate in warmer climates. In Italy, *N*. *ceranae* seems to have completely replaced *N*. *apis* with no *N*. *apis*-positive sample being found in the two year-monitoring study. Supporting data collected in the BeeNet monitoring network found only one sample positive for *N*. *apis*, in an apiary in Bolzano province. The prevalence of *N*. *ceranae* ranged, on average, from 47 to 69% in 2009 and from 30 to 60% in 2010 with a strong seasonality effect. The prevalence of *Nosema*-positive apiaries decreased from spring to autumn in both years. This trend has also been observed in Germany and United States [46, 47]. Overall, the level of *N*. *ceranae* prevalence in Italy was lower than Belgium (92.6%) and Spain (65.6%) but higher than Germany (from 5.2% to 35.4% in 2008) [27, 46, 48].

In general, virus prevalence was higher in 2010 than in 2009. The presence of SBV, ABPV and DWV significantly varied over seasons, showing an increase in level of infection from spring to autumn. A similar trend was also observed for BQCV, but only in 2009. Several viruses (i.e. ABPV, KBV, IAPV, and DWV) which are known to be associated with *Varroa* infestation [49, 50], were found to be in higher prevalence in the late summer-early winter, which could potentially be down to an increased *Varroa* infestation as the season progressed [51]. The five most frequent viruses (DWV, BQCV, SBV, ABPV, CBPV) were found in all Italian regions, with differing abundance. SBV, ABPV and DWV were detected more frequently in the southern regions of Italy. KBV and IAPV were found only in a few regions. Overall, the level of virus infection in Italy was higher than the level reported in Germany and in Belgium [24, 25, 52].

### Bee-bread quality

Previous studies have highlighted the importance of pollen quality for bee health, both at individual [53] and at colony level [54]. In particular, pollen characterized to be of good quality includes high protein content and the presence of the ten essential amino acids [55]. Data showed that the raw protein content in bee-bread declines in the second part of the year (late summer-autumn). This trend has been supported by data collected in the BeeNet monitoring scheme. Our results also indicated that the landscape surrounding the apiaries, in terms of agricultural land use, strongly influences pollen quality.

### Pesticides

The results of the pesticides survey in ApeNet showed contamination of all tested materials, including samples with multiple residues. In bee-bread, 27% of the samples collected were positive. Several investigations throughout Europe revealed higher percentages of contamination in this matrix: 58% in France [23], 42% in Spain [21] and 75% in Germany [25]. This discrepancy could be partially explained by the organisation of the ApeNet network, covering different territories, from intensive agricultural landscapes to natural protected environment, with 45% of samples collected from apiaries located in non-cultivated areas. However, neither macro area, sampling period or year seem to influence the percentage of positive samples. Bee-bread had the highest number of active ingredients detected, when compared with the other two sample matrices (bee-bread: 25 active ingredients; honey bees: 21; beeswax: 17). Relevant amounts of the fungicides (fludioxonil, propamocarb) and of neonicotinoid insecticides (clothianidin, thiamethoxam) were detected. All these active ingredients were present at extremely high concentrations when compared with the available data (see Table 1 in [56] for a review). However, only three active ingredients (clothianidin, imidacloprid and thiamethoxam) were found in bee-bread in concentrations higher than their LD_50_.

Beeswax was the most frequently contaminated material, with 40% of samples testing positive including 12.7% with multiple residues. The most frequent pesticides found were organophosphates and pyrethroids such as coumaphos, tau-fluvalinate, commonly used against *Varroa* mites. This suggests that the low turnover of combs might determine a relevant and persistent contamination of the hive [22]. Comparing maximum pesticide residues found in ApeNet network with the data collected in other monitoring studies (see Table 1 in [54] for a review), we often found higher concentrations. The LD_50_ was exceeded only for fipronil in 2010.

Only 11.7% of honey bee samples were positive for pesticides. In honey bees, like in the other two matrices, anti-varroa compounds were frequently found. An agricultural insecticide, rotenone, gave the highest percentage prevalence in 2009 (35%). When compared with other monitoring surveys (see Table 1 in [54] for a review), we found higher concentrations for bitertanol (119 ng/g), chlorpyrifos ethil (121 ng/g), flumethrin (452 ng/g), clothianidin (103 ng/g) and imidacloprid (86 ng/g). The last two compounds were retrieved in concentration higher than their LD_50_.

### Association between pathogens

In both years the highest number of significant positive associations was observed in the 2^nd^ sampling period (respectively, 6 and 3). Some of the positive associations e.g. ABPV/DWV, ABPV/BQCV and DWV/BQCV were also found in CCD colonies [57]. No significant association was observed between *N*. *ceranae* and the viruses and therefore does not confirm the potential antagonistic effect between *Nosema* and DWV [58, 59].

### Colony losses

The annual colony loss in Italy in 2009 was on average 19.2%. This value seems to be particularly low compared to data recorded in 2007 showing 30–40% of colony losses in the Northern areas and 10–30% in the Central and Southern areas [36]. Similar results were reported in 2008 with a percentage of colony losses ranging from 25 to 38% [36]. Following these high colony losses and the reports of bee mortality in spring during maize sowing, in 2008 the Italian Government banned all four compounds registered for seed dressing—imidacloprid, thiamethoxam, clothianidin and fipronil. Following this decision colony losses and honey bee mortality reports in Italy decreased (BeeNet project: Colony losses 2012: ~12.5% and 2013: ~11.6%—www.reterurale.it/api).

In 2009 and 2010, the colony mortality rate was positively related with the percentage of agricultural areas surrounding the apiaries. This result suggests the importance of a healthy environment, with a high plant biodiversity and low pesticide impact for providing high-quality food sources for the colony. A low-contaminated and diverse landscape has been also demonstrated to reduce the susceptibility of honey bees to other stressors [16, 33]. An effect of land cover types on winter colony losses was also observed in a monitoring study in Luxembourg but, in this case, they found a significant positive relationship between the colony losses and the land use related to leisure, transport and industry, rather an effect of the agricultural area [60].

DWV detection in autumn 2009 is positively correlated with colony losses, in accordance with the outcomes of the German bee monitoring project [25]. Similarly, hive mortality was higher in cases of BQCV infection in the first and second samples of the year. Although we did not assess the *Varroa* infestation, the impact of this pathogen on colony losses may be significant since it acts as biological virus vector and enhances the virulence of some viruses [61]. This result was also in agreement with the data gathered in BeeNet project where a significant association was observed between DWV infection load, *Varroa* infestation and colony losses (data not shown). The presence of ABPV and IAPV was low and not related to the health status of the colonies. Data could not confirm that those two viruses (ABPV and IAPV) are linked to CCD-like symptoms or colony decline, as hypothesised by Cox-Foster et al. [3] and Nguyen et al. [62].

The percentage of colony losses was also higher in apiaries positive to *N*. *ceranae* in the first sampling period. These results support the significant role of this pathogen, even in the early season and the effect on the subsequent development and survival of the colony [63]. However, no relationship between *Nosema* spp, virus infection and colony losses were observed in 2010. In this year, seasonal losses were significantly related to the presence of pesticides in honey bees during the second sampling period. Result shows that pesticide contamination, possibly results in acute poisoning in spring, may have a negative impact at colony level, increasing colony mortality reported during the active season.

## Supporting Information

S1 Protocol Determination of pesticides in bee samples. (DOCX)

S1 Table Municipality and region of the ApeNet apiaries. (DOCX)

S2 Table Primer sequences. (DOCX)

S3 Table Compounds investigated by LC-MS. (DOCX)

S4 Table Compounds investigated by GC-ECD. (DOCX)

S5 Table Percentage of samples positive to *Nosema ceranae* classified per year, macro area and period. (DOCX)

S6 Table Percentage of samples positive for BQCV, SBV, ABPV, CBPV, DWV, KBV, IAPV and AIV classified per year, macro area and period. (DOCX)

S7 Table Percentage of proteins in bee-bread classified per year, macro area and period. (DOCX)

S8 Table Percentage of samples positive for pesticides in three bee matrices: honey bees, bee-bread and beeswax. (DOCX)

S9 Table Number of positive sample (N) for each compound and percentage of pesticide detection (%) in honey bees, beeswax and bee-bread samples from Italian bee colonies in 2009 and 2010. (DOCX)

S10 Table Year 2009. Association between pathogens in sampling periods.
(DOCX)

S11 Table Year 2010. Association between pathogens in sampling periods.
(DOCX)

S12 Table Percentage of colony losses per each monitored Italian region and year (N = number of apiaries per each region). (DOCX)

## References

[1] Aizen MA, Harder LD. The global stock of domesticated honey bees is growing slower than agricultural demand for pollination. Curr Biol 2009; 19: 1–4. 19427214 10.1016/j.cub.2009.03.071

[2] Potts SG, Roberts SPM, Dean R, Marris G, Brown MA, Jones HR, et al Declines of managed honey bees and beekeepers in Europe. J Apic Res 2010; 49, 15–22.

[3] Cox-Foster DL, Conlan S, Holmes EC, Palacios G, Evans JD, Moran NA, et al A metagenomic survey of microbes in honey bee colony collapse disorder. Science 2007; 318, 283–287. 17823314 10.1126/science.1146498

[4] vanEngelsdorp D, Underwood R, Caron D, Hayes J. An estimate of managed colony losses in the winter of 2006–2007: A report commissioned by the apiary inspectors of America. Am Bee J 2007;147, 599–603.

[5] Underwood RM, vanEngelsdorp D. Colony Collapse Disorder: have we seen this before? Bee Culture 2007; 135: 13–18.

[6] vanEngelsdorp D, Evans JD, Saegerman C, Mullin C, Haubruge E, Nguyen BK, et al Colony Collapse Disorder: A Descriptive Study. PLoS ONE 2009; 4: e6481 10.1371/journal.pone.0006481 19649264 PMC2715894

[7] Lee KV, Steinhauer N, Rennich K, Wilson ME, Tarpy DR, Caron DM et al A national survey of managed honey bee 2013–2014 annual colony losses in the USA. Apidologie 2015; 46: 292–305.

[8] Bortolotti L, Sabatini AG, Mutinelli F, Astuti M, Lavazza A, Piro R, et al Spring honey bee losses in Italy. Julius-Kühn Archives 2009; 423: 148–152.

[9] Van der Zee R, Brodschneider R, Brusbardis V, Charrière J-D, Chlebo R, Coffey MF, et al Results of international standardised beekeeper surveys of colony losses for winter 2012–2013: analysis of winter loss rates and mixed effects modelling of risk factors for winter loss. J Apicult Res 2014; 53: 19–34.

[10] Mutinelli F, Costa C, Lodesani M, Baggio A, Medrzycki P, Formato G, et al Honey bee colony losses in Italy. J Apicult Res 2010; 49: 119–120.

[11] Maini S, Medrzycki P, Porrini C. The puzzle of honey bee losses: a brief review. B Insectol 2010; 63: 153–160.

[12] Alaux C, Brunet JL, Dussaubat C, Mondet F, Tchamitchan S, Cousin M, et al Interactions between Nosema microspores and a neonicotinoid weaken honeybees (Apis mellifera). Environ Microbiol 2010; 12: 774–782. 10.1111/j.1462-2920.2009.02123.x 20050872 PMC2847190

[13] Doublet V, Labarussias M, de Miranda JR, Moritz RFA, Paxton RJ. Bees under stress: sublethal doses of a neonicotinoid pesticide and pathogens interact to elevate honey bee mortality across the life cycle. Environ Microbiol 2015; 17: 969–983. 10.1111/1462-2920.12426 25611325

[14] Nazzi F, Brown SP, Annoscia D, Del Piccolo F, Di Prisco G, Varricchio P, et al Synergistic parasite-pathogen interactions mediated by host immunity can drive the collapse of honeybee colonies. PLoS Pathog 2012; 8: e1002735 10.1371/journal.ppat.1002735 22719246 PMC3375299

[15] Pettis J, vanEngelsdorp D, Johnson J, Dively G. Pesticide exposure in honey bee results in increased levels of the gut pathogen Nosema. Naturwissenschaften 2012; 99: 153–158. 10.1007/s00114-011-0881-1 22246149 PMC3264871

[16] Di Prisco G, Cavaliere V, Annoscia D, Varricchio P, Caprio E, Nazzi F, et al Neonicotinoid clothianidin adversely affects insect immunity and promotes replication of a viral pathogen in honey bees. Proc Nat Acad Sci USA 2013; 110: 18466–18471. 10.1073/pnas.1314923110 24145453 PMC3831983

[17] Sgolastra F, Renzi T, Draghetti S, Medrzycki P, Lodesani M, Maini S, et al Effects of neonicotinoid dust from maize seed-dressing on honey bees. B Insectol 2012; 65: 273–280.

[18] David A, Botìas C, Abdul-Sada A, Nicholls E, Rotheray EL, Hill EM, et al Widespread contamination of wildflower and bee-collected pollen with complex mixtures of neonicotinoids and fungicides commonly applied to crops. Environ Int 2016; 88: 169–178. 10.1016/j.envint.2015.12.011 26760714

[19] Ravoet J, Reybroeck W, de Graaf DC. Pesticides for apicultural and/or agricultural application found in Belgian honey bee wax combs. B Environ Contam Tox 2015; 94: 543–548. 10.1007/s00128-015-1511-y PMC4391733 25749505

[20] Chauzat MP, Martel AC, Cougoule N, Porta P, Lachaize J, Zeggane S, et al An Assessment of Honeybee Colony Matrices, Apis Mellifera (Hymenoptera Apidae) to Monitor Pesticide Presence in Continental France. Environ Toxicol Chem 2011; 30: 103–111. 10.1002/etc.361 20853451

[21] Bernal J, Garrido Bailén E, Del Nozal MJ, González-Porto AV, Martín HR, Diego JC, et al Overview of pesticide residues in stored pollen and their potential effect on bee colony (Apis mellifera) losses in Spain. J Econ Entomol 2010; 103: 1964–1971. 21309214 10.1603/ec10235

[22] Mullin CA, Frazier M, Frazier JL, Ashcraft S, Simonds R, van Engelsdorp D, et al High levels of miticides and agrochemicals in North American apiaries: implications for honey bee health. PLoS ONE 2010; 5: e9754 10.1371/journal.pone.0009754 20333298 PMC2841636

[23] Lambert O, Piroux M, Puyo S, Thorin C, L'Hostis M, Wiest L, et al Widespread Occurrence of Chemical Residues in Beehive Matrices from Apiaries Located in Different Landscapes of Western France. PLoS ONE 2013; 8: e67007 10.1371/journal.pone.0067007 23799139 PMC3684584

[24] Simon-Delso N, San Martin G, Bruneau E, Minsart LA, Mouret C, Hautier L. Honeybee Colony Disorder in Crop Areas: The Role of Pesticides and Viruses. PLoS ONE 2014; 9: e103073 10.1371/journal.pone.0103073 25048715 PMC4105542

[25] Genersch E, von der Ohe W, Kaatz H, Schroeder A, Otten C, Buchler R, et al The German bee monitoring project: a long term study to understand periodically high winter losses of honey bee colonies. Apidologie 2010; 41: 332–352.

[26] Berthoud H, Imdorf A, Haueter M, Radloff S, Neumann P. Virus infections and winter losses of honey bee colonies (*Apis mellifera*). J Apicult Res 2010; 49: 60–65.

[27] Ravoet J, Maharramov J, Meeus I, De Smet L, Wenseleers T, Smagghe G, et al Comprehensive Bee Pathogen Screening in Belgium Reveals Crithidia mellificae as a New Contributory Factor to Winter Mortality. PLoS ONE 2013; 8: e72443 10.1371/journal.pone.0072443 23991113 PMC3753275

[28] Bollan KA, Hothersall JD, Moffat C, Durkacz J, Saranzewa N, Wright GA, et al The microsporidian parasites Nosema ceranae and Nosema apis are widespread in honeybee (Apis mellifera) colonies across Scotland. Parasitol Res 2013; 112: 751–759. 10.1007/s00436-012-3195-0 23180128

[29] Botìas C, Martin-Hernandez R, Garrido-Bailon E, Gonzalez-Porto A, Martinez-Salvador A, De La Rua P, et al The growing prevalence of Nosema ceranae in honey bees in Spain, an emerging problem for the last decade. Res Vet Sci 2012; 93: 150–155. 10.1016/j.rvsc.2011.08.002 21906767

[30] Chauzat MP, Carpentier P, Madec F, Bougeard S, Cougoule N, Drajnudel P, et al The role of infectious agents and parasites in the health of honey bee colonies in France. J Apicult Res 2010; 49: 31–39.

[31] Odoux JF, Feuillet D, Aupinel P, Loublier Y, Tasei JN, Mateescu C. Territorial biodiversity and consequences on physico-chemical characteristics of pollen collected by honey bee colonies. Apidologie 2012; 43: 561–575.

[32] Alaux C, Ducloz F, Crauser D, Le Conte Y. Diet effects on honeybee immunocompetence. Biol Lett 2010; 6: 562–565. 10.1098/rsbl.2009.0986 20089536 PMC2936196

[33] Tosi S, Medrzycki P, Bogo G, Bortolotti L, Grillenzoni F, Forlani G. Role of food quality in bee susceptibility to fipronil and clothianidin. Julius-Kühn-Archives 2012; 437: 106.

[34] EC. Commission implementing Regulation No 485/2013 of 24 May 2013 amending Implementing Regulation (EU) No 540/2011, as regards the conditions of approval of the active substances clothianidin, thiamethoxam and imidacloprid, and prohibiting the use and sale of seeds treated with plant protection products containing those active substances. OJ EU 25 5 2013; L139: 12–26.

[35] Greatti M, Barbattini R, Stravisi A, Sabatini AG, Rossi S. Presence of the a.i. imidacloprid on vegetation near corn fields sown with Gaucho^®^ dressed seeds. B Insectol 2006; 59: 99–103.

[36] Mutinelli F, Sgolastra F, Gallina A, Medrzycki P, Bortolotti L, Lodesani M, et al A network for monitoring honeybee mortality and colony losses in Italy as a part of the APENET research project. Am Bee J 2010; 150: 389–390.

[37] Porrini C, Sgolastra F, Sabatini AG. Rete per il monitoraggio dei fenomeni di spopolamento e mortalità degli alveari in Italia (APENET). APOidea 2008; 5: 83–87.

[38] OIE. Nosemosis of honey bees 2008. Chapter 2.2.4 In Manual of Diagnostic Tests and Vaccines for Terrestrial Animals. 6th Edition Paris.

[39] Higes M, Martìn R, Meana A. Nosema ceranae, a new microsporidian parasite in honey bees in Europe J Invertebr Pathol 2006; 92: 93–95. 16574143 10.1016/j.jip.2006.02.005

[40] Webster TC, Pomper KW, Hunt G, Thacker EM, Jones SC. Nosema apis infection in worker and queen Apis mellifera. Apidologie 2004; 35: 49–54.

[41] Martin SJ, Highfield AC, Brettel L, Villalobos EM, Budge GE, Powell M, et al Global honey bee viral landscape altered by a parasitic mite. Science 2012; 336: 1034–1036. 10.1126/science.1220941 22679096

[42] Chantawannakul P, Ward L, Boonham N, Brown MA. A scientific note on the detection of honeybee viruses using real-time PCR (TaqMan) in Varroa mites collected from a Thai honeybee (Apis mellifera) apiary. J Invertebr Pathol 2006; 91: 69–73. 16376930 10.1016/j.jip.2005.11.001

[43] Kajobe R, Marris G, Budge G, Laurenson L, Cordoni G, Jones B, et al First molecular detection of a viral pathogen in Ugandan honey bees. J Invertebr Pathol 2010; 104: 153–156. 10.1016/j.jip.2010.02.007 20219470

[44] Zar JH. Biostatistical analysis. Biostatistical Analysis. Prentice-Hall, Upper Saddle River, New Jersey, 663 pp; 1999.

[45] Fries I. Nosema ceranae in European honey bees (Apis mellifera). J Invertebr Pathol 2010; 103: S73–S79. 10.1016/j.jip.2009.06.017 19909977

[46] Gisder S, Hedtke K, Mockel N, Frielitz MC, Linde A, Genersch E. Five-Year Cohort Study of Nosema spp. in Germany: Does Climate Shape Virulence and Assertiveness of Nosema ceranae? Appl Environ Microb 2010; 76: 3032–3038. 10.1128/AEM.03097-09 PMC2863439 20228103

[47] Traver BE, Williams MR, Fell RD. Comparison of within hive sampling and seasonal activity of Nosema ceranae in honey bee colonies. J Invertebr Pathol 2012; 109: 187–193. 10.1016/j.jip.2011.11.001 22085836

[48] Higes M, Martín-Hernández R, Martínez-Salvador A, Garrido-Bailón E, González-Porto AV, Meana A, et al A preliminary study of the epidemiological factors related to honey bee colony loss in Spain. Environ Microbiol Rep 2010; 2: 243–250. 10.1111/j.1758-2229.2009.00099.x 23766075

[49] de Miranda JR, Cordoni G, Budge G. The acute bee paralysis virus–Kashmir bee virus–Israeli acute paralysis virus complex. J Invert Pathol 2010; 103: S30–S47. 10.1016/j.jip.2009.06.014 19909972

[50] de Miranda JR, Genersch E. Deformed wing virus. J Invert Pathol 2010; 103: S48–S61. 10.1016/j.jip.2009.06.012 19909976

[51] Ball BV, Allen MF. The Prevalence of Pathogens in Honey Bee (Apis-Mellifera) Colonies Infested with the Parasitic Mite Varroa-Jacobsoni. Ann Appl Biol 1988; 113: 237–244.

[52] De Smet L, Ravoet J, de Miranda JR, Wenseleers T, Mueller MY, Moritz RFA, et al BeeDoctor, a Versatile MLPA-Based Diagnostic Tool for Screening Bee Viruses. PLoS ONE 2012; 7: e47953 10.1371/journal.pone.0047953 23144717 PMC3483297

[53] Di Pasquale G, Salignon M, Le Conte Y, Belzunces LP, Decourtye A, Kretzschmar A, et al Influence of Pollen Nutrition on Honey Bee Health: Do Pollen Quality and Diversity Matter? PLoS ONE 2013; 8: e72016 10.1371/journal.pone.0072016 23940803 PMC3733843

[54] Brodschneider R, Crailsheim K. Nutrition and health in honey bees. Apidologie 2010; 41: 278–294.

[55] Roulston TH, Cane JH. Pollen nutritional content and digestibility for animals. Plant Syst Evol 2000; 222: 187–209.

[56] Johnson RM, Ellis MD, Mullin CA, Frazier M. Pesticides and honey bee toxicity—USA. Apidologie 2010; 41: 312–331.

[57] Cornman RS, Tarpy DR, Chen Y, Jeffreys L, Lopez D, Pettis JS, et al Pathogen Webs in Collapsing Honey Bee Colonies. PLoS ONE 2012; 7: e43562 10.1371/journal.pone.0043562 22927991 PMC3424165

[58] Costa C, Tanner G, Lodesani M, Maistrello L, Neumann P. Negative correlation between Nosema ceranae spore loads and deformed wing virus infection levels in adult honey bee workers. J Invertebr Pathol 2011; 108: 224–225. 10.1016/j.jip.2011.08.012 21939664

[59] Doublet V, Natsopoulou ME, Zschiesche L, Paxton RJ. Within-host competition among the honey bees pathogens Nosema ceranae and Deformed wing virus is asymmetric and to the disadvantage of the virus. J Invertebr Pathol 2015; 124: 31–34. 10.1016/j.jip.2014.10.007 25450738

[60] Clermont A, Eickermann M, Kraus F, Hoffmann L, Beyer M. Correlations between land covers and honey bee colony losses in a country with industrialized and rural regions. Science of Total Environment 2015; 532: 1–13. 10.1016/j.scitotenv.2015.05.128 26057621

[61] McMenamin AJ, Genersch E. Honey bee colony losses and associated viruses. Current Opinion in Insect Science 2015; 8: 121–129. 10.1016/j.cois.2015.01.015 32846659

[62] Nguyen BK, Ribière M, VanEngelsdorp D, Snoek C, Saegerman C, Kalkstein AL, et al Effects of honey bee virus prevalence, Varroa destructor load and queen condition on honey bee colony survival over the winter in Belgium. J Apicult Res 2011; 50: 195–202.

[63] Higes M, Martin-Hernandez R, Garrido-Bailon E, Gonzalez-Porto AV, Garcia-Palencia P, Meana A, et al Honeybee colony collapse due to Nosema ceranae in professional apiaries. Env Microbiol Rep 2009; 1: 110–113. 23765741 10.1111/j.1758-2229.2009.00014.x

