# Supplementary material for: The Status of Honey Bee Health in Italy: Results from the Nationwide Bee Monitoring Network
Source: PLoS One. 2016 May 16;11(5):e0155411. doi: 10.1371/journal.pone.0155411 (PMC4868308; doi:10.1371/journal.pone.0155411)
Supplement: S1 Protocol — (DOCX) [file pone.0155411.s001.docx]

**S1 Protocol. Determination of pesticides in bee samples.**

**Reagents and Standards**

All the solvent and chemicals used in this study were of analytical reagent grade; acetonitrile (MeCN), formic acid, ammonium formiate, methanol, acetic acid (AcOH) and heptane (Sigma Aldrich). Distilled water was purified at 18.2 MΩ with a MilliQ ULTRA (Millipore). Quechers Extraction Tube EN Method (4 g magnesium sulphate, 1g sodium chloride, 0.5 g sodium hydrogencitrate sesquihydrate, and 1 g sodium citrate), Dispersive SPE Fatty Samples (900 mg Magnesium sulphate, 150 mg PSA and 150 mg C18), Dispersive SPE Hight Pigment (150 mg PSA, 45 mg GCB, 855 mg magnesium sulphate) were used (Agilent Tecnologies). The certified analytical standards were supplied from Sigma- Aldrich and most of them were of > 99% certified purity. Concentrations of standard solutions were corrected by the certified purity of the standards, whenever below 99%. Individual stock standard solutions of each pesticide were prepared by dissolving the appropriate amount of each compound in 10 ml of acetone to prepare a 1000 µg/ml stock solution.

A multicompound intermediate standard solution at a concentration of 10 µg/mL and 1 µg/mL were prepared via appropriate dilutions of the stock solutions in MeCN containing 0.1% AcOH in order to prevent degradation of the analytes for LC-MS analysis, while for GC analysis multicompound intermediate standard solution were prepared in heptane.

The individual stock solution, mixed intermediate and working standard solutions were stored at -20°C in dark amber bottles for a maximum period of 6 months.

**Instruments**

A Prominence series system (Shimadzu) consisting of two LC-20AD XR pumps, an SIL-20A XR autosampler, a DGU-20A3 vacuum degasser and a CTO-20. A column oven was used for chromatographic separations. The analytical column for the separation of the analytes was a RP- Amide Ascentis Express (10 cm x 2.1 mm, 2.7 µm) with adequate guard column maintained at 40 °C. The separation of the analytes was performed by applying a gradient of components A (5 mM ammonium formiate in water with 0.1% formic acid) and B (Methanol whit 5 mM ammonium formiate and 0.1% formic acid) with a flow rate of 0.4 mL min^-1^. The injector volume was 10 µL.The gradient started with 5% of component B for 1 min and then increase to 95% within 16 min. This composition was kept for 4 min, then decrease to 5% component B within 1 min. With the following equilibration time of 3 min, the resulting total run time was 25 min.

A LCMS-2010 EV single quadrupole mass spectrometer (Shimadzu), equipped with an electrospray ionization (ESI) interface was used for the detection and quantification of pesticides. MS parameters were as follows: ESI interface voltage 1.5 kV, CDL temperature 250°C and the clock heater temperature 200°C. Nitrogen was the nebulising gas with a flow rate of 1.5 L min^-1^. Drying gas pressure was set at 0.1 MPa.

Gas chromatography analyses were performed with a gas chromatograph GC 2014 Shimatzu equipped with an HT300A HTA autosampler, a split/splitness injector and two ^63^Ni electron capture detector. A 5 m integrated guard column was coupled to a Restek RTX-CLPesticides (30 m ×0, 25 mm×0.25 µm) and a Restek RTX-CLPesticides2 (30 m ×0.25 mm×0.20 µm). A sample volume of 0.5 µL was injected into the split mode at an injector temperature of 120°C. The oven temperature program was as follows: initial temperature 60 °C (held for 2 mins) increased by 70°C/min to 200°C; increased by 2°C /min to 300°C (held for 1 min); increased by 40°C/min to 310°C and held for 1 min. The detector temperature was held at 320°C. Nitrogen was served as make up gas with a flow rate of 3 mL/min. The concentration of the calibration standards were 100 and 200 µg/L.

**Sample extraction and clean-up**

A representative portion of sample was processed using a homogenizer with liquid nitrogen, transferred into plastic bags and stored at -20°C prior to analysis. The homogenized sample of bees (2 g) was weighed into a 50 ml polypropylene centrifuge tube, 10 ml of water and 10 ml of acetonitrile with 0.1 % acetic acid (v/v) were added and the mixture was shaken vigorously (2000 rpm) for 10 min. The sample was placed in a freezer at the temperature of -20°C for a minimum of 2 h. Ceramic homogenizers, magnesium sulphate (4 g), sodium chloride (1 g), sodium hydrogencitrate sesquihydrate (0.5 g) and sodium citrate (1 g) were added to the mixture and immediately shaken (3000 rpm) for 1 min. followed by centrifugation (4500 rpm) at -20°C for 10 min.

After centrifugation, 6 ml of supernatant was transferred into a 15 mL tube containing 900 mg magnesium sulphate, 150 mg PSA and 150 mg C18 and was stirred for 1 min (2000 rpm) followed by centrifuged (4500 rpm) at -20°C for 5 min. Finally, an aliquot of supernatant was transferred to clean tube and the extract reduced to dryness using a nitrogen evaporator at 40°C. The residue was redissolved with 1 ml of heptane for GC analysis or with 500 μL of 5 mM ammonium formiate in water with 0.1% formic acid / Methanol with 5 mM ammonium formiate and 0.1% formic acid (50/50, v/v).

**Chemical Analyses – Nitrogen content in bee-bread**

Digestion was performed in the Kjeldatherm block with fume removal system (Gerhardt), distillation and titration in the Vapodest apparatus (Gerhardt). For mineralisation sulphuric acid (96%, Carlo Erba) and Kjeltabs Cu/3.5 (0.4 g CuSO_4_×5 H_2_O, 3.5 g K_2_SO_4_, Foss Italia) were used. For distillation NaOH (32% w/v, Titolchimica) and boric acid (4% w/v, Titolchimica) were used. For titration HCL (0.1 N, Titolchimica) was used.
