# Supplementary material for: The Status of Honey Bee Health in Italy: Results from the Nationwide Bee Monitoring Network
Source: PLoS One. 2016 May 16;11(5):e0155411. doi: 10.1371/journal.pone.0155411 (PMC4868308; doi:10.1371/journal.pone.0155411)
Supplement: S1 Table — (DOCX) [file pone.0155411.s002.docx]

**S1 Table. Municipality and region of the ApeNet apiaries.**

| **N** | **Module code** | **Apiary code** | **Municipality** | **Region** | **Elevation (m)** |
| --- | --- | --- | --- | --- | --- |
| 1 | ABR1 | ABR1-1 | Palombaro | Abruzzo | 400 |
| 2 | ABR1 | ABR1-2 | Montesilvano | Abruzzo | 10 |
| 3 | ABR1 | ABR1-3 | Scontrone | Abruzzo | 850 |
| 4 | ABR1 | ABR1-4 | Teramo | Abruzzo | 230 |
| 5 | ABR1 | ABR1-5 | L'Aquila | Abruzzo | 700 |
| 6 | ABR1 | ABR1-6 | Teramo | Abruzzo | 530 |
| 7 | BLZ1 | BLZ1-1 | San Genesio Atesino | Trentino Alto Adige | 850 |
| 8 | BLZ1 | BLZ1-2 | Lana | Trentino Alto Adige | 350 |
| 9 | BLZ1 | BLZ1-3 | Malles Venosta | Trentino Alto Adige | 1150 |
| 10 | BLZ1 | BLZ1-4 | Valle Di Casies | Trentino Alto Adige | 1200 |
| 11 | BSL1 | BSL1-1 | Pignola | Basilicata | 970 |
| 12 | BSL1 | BSL1-2 | Melfi | Basilicata | 580 |
| 13 | BSL1 | BSL1-3 | Paterno | Basilicata | 620 |
| 14 | BSL1 | BSL1-4 | Matera | Basilicata | 400 |
| 15 | BSL1 | BSL1-5 | Nova Siri | Basilicata | 254 |
| 16 | CLB1 | CLB1-1 | Crotone | Calabria | 50 |
| 17 | CLB1 | CLB1-2 | Rossano | Calabria | 200 |
| 18 | CLB1 | CLB1-3 | Cropani | Calabria | 50 |
| 19 | CLB1 | CLB1-4 | Petilia Policastro | Calabria | 310 |
| 20 | CLB1 | CLB1-5 | Belvedere Di Spinello | Calabria | 60 |
| 21 | CLB1 | CLB1-6 | Crucoli | Calabria | 43 |
| 22 | CLB2 | CLB2-1 | Catanzaro | Calabria | 53 |
| 23 | CLB2 | CLB2-2 | Amaroni | Calabria | 243 |
| 24 | CLB2 | CLB2-3 | San Sostene | Calabria | 4 |
| 25 | CLB2 | CLB2-4 | Lamezia Terme | Calabria | 200 |
| 26 | CLB2 | CLB2-5 | Sersale | Calabria | 578 |
| 27 | CLB3 | CLB3-1 | Cosenza | Calabria | 480 |
| 28 | CLB3 | CLB3-2 | Acri | Calabria | 430 |
| 29 | CLB3 | CLB3-3 | Rossano | Calabria | 80 |
| 30 | CLB3 | CLB3-4 | Villlapiana | Calabria | 70 |
| 31 | CLB3 | CLB3-5 | Mottafollone | Calabria | 400 |
| 32 | CLB4 | CLB4-1 | Grotteria | Calabria | 250 |
| 33 | CLB4 | CLB4-2 | Reggio di Calabria | Calabria | 100 |
| 34 | CLB4 | CLB4-3 | Careri | Calabria | 50 |
| 35 | CLB4 | CLB4-4 | Gioia Tauro | Calabria | 50 |
| 36 | CLB4 | CLB4-5 | Melito di Porto Salvo | Calabria | 50 |
| 37 | CMP1 | CMP1-1 | Nola | Campania | 347 |
| 38 | CMP1 | CMP1-2 | Giugliano In Campania | Campania | 3 |
| 39 | CMP1 | CMP1-3 | Agropoli | Campania | 4 |
| 40 | CMP1 | CMP1-4 | Castel Volturno | Campania | 2 |
| 41 | CMP1 | CMP1-5 | Giffoni Valle Piana | Campania | 254 |
| 42 | EMR1 | EMR1-1 | Agazzano | Emilia Romagna | 108 |
| 43 | EMR1 | EMR1-2 | Reggio Nell'Emilia | Emilia Romagna | 53 |
| 44 | EMR1 | EMR1-3 | Ozzano Dell'Emilia | Emilia Romagna | 94 |
| 45 | EMR1 | EMR1-4 | Molinella | Emilia Romagna | 5 |
| 46 | EMR1 | EMR1-5 | Casola Valsenio | Emilia Romagna | 165 |
| 47 | EMR2 | EMR2-1 | Sasso Marconi | Emilia Romagna | 370 |
| 48 | EMR2 | EMR2-2 | Anzola Dell'Emilia | Emilia Romagna | 35 |
| 49 | EMR2 | EMR2-3 | Sala Bolognese | Emilia Romagna | 20 |
| 50 | EMR2 | EMR2-4 | Monterenzio | Emilia Romagna | 400 |
| 51 | EMR2 | EMR2-5 | Granaglione | Emilia Romagna | 1050 |
| 52 | EMR3 | EMR3-1 | Gragnano Trebbiense | Emilia Romagna | 80 |
| 53 | EMR3 | EMR3-2 | Fiorenzuola d'Arda | Emilia Romagna | 62 |
| 54 | EMR3 | EMR3-3 | Albinea | Emilia Romagna | 253 |
| 55 | EMR3 | EMR3-4 | Soliera | Emilia Romagna | 23 |
| 56 | EMR3 | EMR3-5 | Bazzano | Emilia Romagna | 55 |
| 57 | EMR4 | EMR4-1 | Malalbergo | Emilia Romagna | 11 |
| 58 | EMR4 | EMR4-2 | Dozza | Emilia Romagna | 79 |
| 59 | EMR4 | EMR4-3 | Jolanda di Savoia | Emilia Romagna | 15 |
| 60 | EMR4 | EMR4-4 | Civitella di Romagna | Emilia Romagna | 388 |
| 61 | EMR4 | EMR4-5 | Cervia | Emilia Romagna | 9 |
| 62 | LGR1 | LGR1-1 | Airole | Liguria | 450 |
| 63 | LGR1 | LGR1-2 | Triora | Liguria | 900 |
| 64 | LGR1 | LGR1-3 | San Remo | Liguria | 150 |
| 65 | LGR1 | LGR1-4 | Savona | Liguria | 150 |
| 66 | LGR1 | LGR1-5 | Piana Crixia | Liguria | 250 |
| 67 | LGR1 | LGR1-6 | Cisano sul Neva | Liguria | 100 |
| 68 | LZO1 | LZO1-1 | Roma | Lazio | 1 |
| 69 | LZO1 | LZO1-2 | Magliano Romano | Lazio | 262 |
| 70 | LZO1 | LZO1-3 | Formello | Lazio | 114 |
| 71 | LZO1 | LZO1-4 | Formello | Lazio | 46 |
| 72 | LZO1 | LZO1-5 | Velletri | Lazio | 369 |
| 73 | MLS1 | MLS1-1 | Agnone | Molise | 788 |
| 74 | MLS1 | MLS1-2 | Fossalto | Molise | 515 |
| 75 | MLS1 | MLS1-3 | Cercemaggiore | Molise | 655 |
| 76 | MLS1 | MLS1-4 | Lupara | Molise | 505 |
| 77 | MLS1 | MLS1-5 | Termoli | Molise | 49 |
| 78 | MRC1 | MRC1-1 | Jesi | Marche | 57 |
| 79 | MRC1 | MRC1-2 | Agugliano | Marche | 282 |
| 80 | MRC1 | MRC1-3 | Falerone | Marche | 414 |
| 81 | MRC1 | MRC1-4 | Tolentino | Marche | 308 |
| 82 | MRC1 | MRC1-5 | Fratte Rosa | Marche | 314 |
| 83 | PGL1 | PGL1-1 | Corato | Puglia | 327 |
| 84 | PGL1 | PGL1-2 | Ceglie Messapico | Puglia | 107 |
| 85 | PGL1 | PGL1-3 | Lecce | Puglia | 35 |
| 86 | PGL1 | PGL1-4 | Martina Franca | Puglia | 432 |
| 87 | PGL1 | PGL1-5 | Gioia Del Colle | Puglia | 82 |
| 88 | SCL1 | SCL1-1 | Lascari | Sicilia | 8 |
| 89 | SCL1 | SCL1-2 | San Vito Lo Capo | Sicilia | 64 |
| 90 | SCL1 | SCL1-3 | Montemaggiore Belsito | Sicilia | 194 |
| 91 | SCL1 | SCL1-4 | Castelvetrano | Sicilia | 50 |
| 92 | SCL1 | SCL1-5 | Monte Pellegrino (Palermo) | Sicilia | 402 |
| 93 | SRD1 | SRD1-1 | Sassari | Sardegna | 99 |
| 94 | SRD1 | SRD1-2 | Oschiri | Sardegna | 643 |
| 95 | SRD1 | SRD1-3 | Sassari | Sardegna | 68 |
| 96 | SRD1 | SRD1-4 | Nurri | Sardegna | 425 |
| 97 | SRD1 | SRD1-5 | Villaspeciosa | Sardegna | 13 |
| 98 | SRD1 | SRD1-6 | Nurri | Sardegna | 613 |
| 99 | TRN1 | TRN1-1 | Levico Terme | Trentino Alto Adige | 487 |
| 100 | TRN1 | TRN1-2 | Stenico | Trentino Alto Adige | 679 |
| 101 | TRN1 | TRN1-3 | Trento | Trentino Alto Adige | 472 |
| 102 | TRN1 | TRN1-4 | Cles | Trentino Alto Adige | 649 |
| 103 | TRN1 | TRN1-5 | Lomaso | Trentino Alto Adige | 764 |
| 104 | TRN1 | TRN1-6 | Baselga di Pinè | Trentino Alto Adige | 983 |
| 105 | TSC1 | TSC1-1 | Prato | Toscana | 139 |
| 106 | TSC1 | TSC1-2 | Montaione | Toscana | 454 |
| 107 | TSC1 | TSC1-3 | San Casciano In Val Di Pe | Toscana | 304 |
| 108 | TSC1 | TSC1-4 | Borgo San Lorenzo | Toscana | 257 |
| 109 | TSC1 | TSC1-5 | Capraia E Limite | Toscana | 134 |
| 110 | TSC2 | TSC2-1 | Capannori | Toscana | 39 |
| 111 | TSC2 | TSC2-2 | Bagni Di Lucca | Toscana | 436 |
| 112 | TSC2 | TSC2-3 | Crespina | Toscana | 43 |
| 113 | TSC2 | TSC2-4 | Pontremoli | Toscana | 299 |
| 114 | TSC2 | TSC2-5 | Livorno | Toscana | 71 |
| 115 | TSC2 | TSC2-6 | Podenzanza | Toscana | 193 |
| 116 | UMB1 | UMB1-1 | Gubbio | Umbria | 407 |
| 117 | UMB1 | UMB1-2 | Assisi | Umbria | 221 |
| 118 | UMB1 | UMB1-3 | Spoleto | Umbria | 282 |
| 119 | UMB1 | UMB1-4 | Marsciano | Umbria | 422 |
| 120 | UMB1 | UMB1-5 | Perugia | Umbria | 203 |
| 121 | UMB1 | UMB1-6 | Todi | Umbria | 152 |
| 122 | UMB2 | UMB2-1 | Castiglione Del Lago | Umbria | 321 |
| 123 | UMB2 | UMB2-2 | Umbertide | Umbria | 279 |
| 124 | VNT1 | VNT1-1 | Bassano Del Grappa | Veneto | 200 |
| 125 | VNT1 | VNT1-2 | Montecchia Di Crosara | Veneto | 90 |
| 126 | VNT1 | VNT1-3 | Noventa Di Piave | Veneto | 5 |
| 127 | VNT1 | VNT1-4 | Nervesa Della Battaglia | Veneto | 100 |
| 128 | VNT1 | VNT1-5 | Conselve | Veneto | 2 |
| 129 | VNT2 | VNT2-1 | Sospirolo | Veneto | 515 |
| 130 | VNT2 | VNT2-2 | Villamarzana | Veneto | 3 |
