# Supplementary material for: The Status of Honey Bee Health in Italy: Results from the Nationwide Bee Monitoring Network
Source: PLoS One. 2016 May 16;11(5):e0155411. doi: 10.1371/journal.pone.0155411 (PMC4868308; doi:10.1371/journal.pone.0155411)
Supplement: S2 Table — (DOCX) [file pone.0155411.s003.docx]

**S2 Table. Primer sequences.**

| Target | Primer Name | Sequence (5’-3’) | Reference |
| --- | --- | --- | --- |
| *N.apis* | NOS-F | TGCCGACGATGTGATATGAG | 39 |
|  | NOS-R | CACAGCATCCATTGAAAACG |  |
| *N.apis* | NosA-F | CCG ACG ATG TGA TAT GAG ATG | 40 |
|  | NosA-R | CAC TAT TAT CAT CCT CAG ATC ATA |  |
| EF1 | EF 1 F | CTGGTACCTCTCAGGCTGATTGT | 41 |
|  | EF 1R | GCATGCTCACGAGTTTGTCCATTCT |  |
|  | EF 1 | TGCTTCGAACTCTCTCCAGTACCAGCAG |  |
| DWV | DWV 9587F | CCT GGA CAA GGT CTC GGT AGA A | 42 |
|  | DWV 9711R | ATT CAG GAC CCC ACC CAA AT |  |
|  | DWV 9627T | CAT GCT CGA GGA TTG GGT CGT CGT |  |
| KBV | KBV 83F | ACC AGG AAG TAT TCC CAT GGT AAG | 42 |
|  | KBV 161R | TGG AGC TAT GGT TCC GTT CAG |  |
|  | KBV 109T | CCG CAG ATA ACT TAG GAC CAG ATC AAT CAC A |  |
| ABPV | APV 95F | TCC TAT ATC GAC GAC GAA AGA CAA | 42 |
|  | APV 159R | GCG CTT TAA TTC CAT CCA ATT GA |  |
|  | APV 121T (MGB) | TTT CCC CGG ACT TGA C |  |
| IAPV | IAPV B4S0427_R130M | RCR TCA GTC GTC TTC CAG GT | 43 |
|  | IAPV B4S0427_L17M | CGA ACT TGG TGA CTT GAR GG |  |
|  | IAPV Probe | TTG CGG CAA TCC AGC CGT GAA AC |  |
| BQCV | BQCV 9195F | GGT GCG GGA GAT GAT ATG GA | 42 |
|  | BQCV 8265R | GCC GTC TGA GAT GCA TGA ATA C |  |
|  | BQCV 8217T | TTT CCA TCT TTA TCG GTA CGC CGC C |  |
| SBV | SBV 311F | AAG TTG GAG GCG CGy AAT TG | 42 |
|  | SBV 380R | CAA ATG TCT TCT TAC dAG AGG yAA GGA TTG |  |
|  | SBV 331T (MGB) | CGG AGT GGA AAG AT |  |
| AIV | AIV 12F | GGC TAG TAA ACG TAG TGG ATA TGA CAA T | 42 |
|  | AIV 106R | CAC CTG GTG GTC CAA GAG AAG |  |
|  | AIV 41T | TGA TTG GAA ATA TAT CTT CTT TAA TAA ACC CAG TTG CTC C |  |
| CPV | CPV 304F | TCT GGC TCT GTC TTC GCA AA | 42 |
|  | CPV 371R | GAT ACC GTC GTC ACC CTC ATG |  |
|  | CPV 325T | TGC CCA CCA ATA GTT GGC AGT CTG C |  |
