# Supplementary material for: The Status of Honey Bee Health in Italy: Results from the Nationwide Bee Monitoring Network
Source: PLoS One. 2016 May 16;11(5):e0155411. doi: 10.1371/journal.pone.0155411 (PMC4868308; doi:10.1371/journal.pone.0155411)
Supplement: S3 Table — (DOCX) [file pone.0155411.s004.docx]

**S3 Table. Compounds investigated by LC-MS.**

| **Compound** | **Class** |
| --- | --- |
| PYRIMETHANIL | anilinopyrimidines |
| CYPRODINIL | anilinopyrimidines |
| CYMOXANIL | acetamides |
| TEFLUBENZURON | benzoiluree |
| TRIFLUMURON | benzoiluree |
| THIABENDAZOLE | benzimidazole |
| THIOPHANATE METHYL | benzimidazole |
| DIFLUBENZURON | benzoiluree |
| ALDICARB | carbamate |
| ALDICARB SULFONE | carbamate |
| ALDICARB SULFOXIDE | carbamate |
| CARBARYL | carbamate |
| CARBOFURAN | carbamate |
| CARBOFURAN 3 IDROSSI | carbamate |
| CARBOFURAN 3 KETO | carbamate |
| DESMETHYL-PIRIMICARB | carbamate |
| FENOTHIOCARB | carbamate |
| FORMETANATE HYDROCLORIDE | carbamate |
| IPROVALICARB | carbamate |
| METHIOCARB SULFOXIDE | carbamate |
| METHOMYL | carbamate |
| OXAMYL | carbamate |
| PIRIMICARB | carbamate |
| PROPAMOCARB | carbamate |
| THIODICARB | carbamate |
| HEXYTHIAZOX | carboxamide |
| BOSCALID | carboxamide |
| TEPRALOXYDIM | cicloesenone |
| TEBUFENOZIDE | diacilidrazine |
| BENALAXYL | phenylamide |
| METALAXYL-M | phenylamide |
| PENCICURON | phenylurea |
| FENPYROXIMATE | fenossipirazole |
| FENHEXAMID | idrossianilide |
| IMAZALIL | imidazolico |
| PROCLORAZ | imidazolico |
| FENAMIDONE | imidazolinoni |
| AZOXYSTROBIN | metossiacrilato |
| DIMETHOMORPH MIXTURE | morfiline |
| DODEMORPH | morfiline |
| FENPROPIMORPH | morfoline |
| ACETAMIPRID | neonicotinoid |
| DINOTEFURAN | neonicotinoid |
| NYTEMPIRAM | neonicotinoid |
| THIACLOPRID | neonicotinoid |
| THIAMETHOXAM | neonicotinoid |
| CLOTHIANIDIN | neonicotinoid |
| IMIDACLOPRID | neonicotinoid |
| DIMETHOAT | organophosphate |
| FENAMIPHOS | organophosphate |
| FENAMIPHOS-SULFON | organophosphate |
| FENAMIPHOS-SULFOXIDE | organophosphate |
| FENITROTHION | organophosphate |
| MALATHION | organophosphate |
| MALOXON | organophosphate |
| OMETHOATE | organophosphate |
| PIRIMIPHOS METHYL | organophosphate |
| CARBOXIN | oxathiin |
| FENARIMOL | piperidine |
| FENPROPIDIN | piperidine |
| TEBUFENPIRAD | pyrazoles carbossimidi |
| ETOFENPROX | pyrethroid |
| TEFLUTRIN | pyrethroid |
| PYRIDABEN | pyridazinone |
| BUPIRIMATE | pyrimidine-idrossipirimidine |
| CHLORMEQUATCHLORID | quaternary ammonium |
| FENAZAQUIN | quinazoline |
| FLAZASULFURON | sulfonylureas |
| IMAZOSULFURON | sulfonylureas |
| PYRACLOSTROBIN | strobilurin |
| CLOFENTEZIN | tetrazine |
| DITHIANON | tiocianochinoni |
| METAMITRON | triazinone |
| BITERTANOL | triazole |
| CYPROCONAZOL | triazole |
| TEBUCONAZOLE | triazole |
| TRIADIMENOL | triazole |
| TRITICONAZOLO | triazole |
| FENBUCONAZOLE | triazole |
| FLUTRIAFOL | triazole |
| PIPERONIL BUTOSSIDO | synergist |
| ROTENONE | rotenone |

This method is characterised by a LOD of 5 ng/g for each analyte.
