# Supplementary material for: The Status of Honey Bee Health in Italy: Results from the Nationwide Bee Monitoring Network
Source: PLoS One. 2016 May 16;11(5):e0155411. doi: 10.1371/journal.pone.0155411 (PMC4868308; doi:10.1371/journal.pone.0155411)
Supplement: S4 Table — (DOCX) [file pone.0155411.s005.docx]

**S4 Table. Compounds investigated by GC-ECD.**

| **Compound** | **Class** |
| --- | --- |
| CAPTAN | carboxamide |
| CYAZOFAMID | cyanoimidazole |
| ETHOPROPHOS | organophosphate |
| ETRIDIAZOL | thiazole |
| FENPROPATRIN | pyrethroid |
| FLUAZINAM | phenyl-pyridinamine |
| FLUQUINCONAZOLE | triazole |
| FOLPET | phthalimide |
| INDOXACARB | oxadiazines |
| IPRODIONE | dicarboximide |
| KRESOXIM METHYL | oximinoacetate |
| METRAFENONE | benzophenone |
| MYCLOBUTANIL | triazole |
| PENCONAZOLO | triazole |
| PROCYMIDONE | dicarboximide |
| TOLCLOFOS METHYL | tiofosfato |
| TRIFLOXYSTROBIN | oxiaminoacetate |
| BIFENTHRIN | pyrethroid |
| CHLORPYRIFOS | organophosphate |
| CHLORPYRIFOS - METHYL | organophosphate |
| LAMBDA - CYHALOTHRIN | pyrethroid |
| CYPERMETHRIN | pyrethroid |
| DELTAMETHRIN | pyrethroid |
| ENDOSULFAN SULFATE | chlorinated |
| BETA ENDOSULFAN | chlorinated |
| ALPHA ENDOSULFAN | chlorinated |
| ESFENVALERATE | pyrethroid |
| FIPRONIL | phenylpyrazole |
| FLUVALINATE | pyrethroid |
| COUMAPHOS | organophosphate |
| ACRINATHRIN | pyrethroid |
| FLUMETRIN | pyrethroid |
| CHLORPHENVINFOS | organophosphate |
| PERMETHRIN MIXTURE | pyrethroid |
| β-CYFLUTHRIN | pyrethroid |
| FENVALERATE | pyrethroid |
| CHLORPROPHAM | carbamate |
| FLUMETHRIN | pyrethroid |
| PENDIMETHALIN | dinitroaniline |

This method is characterised by a LOD of 5 ng/g for each analyte.
