# Supplementary material for: The Status of Honey Bee Health in Italy: Results from the Nationwide Bee Monitoring Network
Source: PLoS One. 2016 May 16;11(5):e0155411. doi: 10.1371/journal.pone.0155411 (PMC4868308; doi:10.1371/journal.pone.0155411)
Supplement: S5 Table — (DOCX) [file pone.0155411.s006.docx]

**S5 Table. Percentage of samples positive to *Nosema ceranae* classified per year, macro area and period.**

| **Year** | **Macro-area** | **Period** | **N** | **%** |
| --- | --- | --- | --- | --- |
| 2009 | N | 1 | 28 | 78.6 |
| 2009 | N | 2 | 28 | 71.4 |
| 2009 | N | 3 | 27 | 51.9 |
| 2009 | N | 4 | 15 | 26.7 |
| 2009 | C | 1 | 20 | 50.0 |
| 2009 | C | 2 | 27 | 48.1 |
| 2009 | C | 3 | 26 | 38.5 |
| 2009 | C | 4 | 14 | 57.1 |
| 2009 | S | 1 | 36 | 63.9 |
| 2009 | S | 2 | 35 | 71.4 |
| 2009 | S | 3 | 34 | 55.9 |
| 2009 | S | 4 | 24 | 50.0 |
| 2010 | N | 1 | 28 | 75.0 |
| 2010 | N | 2 | 26 | 57.7 |
| 2010 | N | 3 | 27 | 40.7 |
| 2010 | N | 4 | 13 | 30.8 |
| 2010 | C | 1 | 24 | 45.8 |
| 2010 | C | 2 | 22 | 36.4 |
| 2010 | C | 3 | 24 | 37.5 |
| 2010 | C | 4 | 14 | 28.6 |
| 2010 | S | 1 | 43 | 58.1 |
| 2010 | S | 2 | 42 | 35.7 |
| 2010 | S | 3 | 43 | 25.6 |
| 2010 | S | 4 | 26 | 23.1 |

N=Northern Italy, C=Central Italy; S=Southern Italy
