# Supplementary material for: The Status of Honey Bee Health in Italy: Results from the Nationwide Bee Monitoring Network
Source: PLoS One. 2016 May 16;11(5):e0155411. doi: 10.1371/journal.pone.0155411 (PMC4868308; doi:10.1371/journal.pone.0155411)
Supplement: S6 Table — (DOCX) [file pone.0155411.s007.docx]

**S6 Table. Percentage of samples positive for BQCV, SBV, ABPV, CBPV, DWV, KBV, IAPV and AIV classified per year, macro area and period.**

| **Year** | **Macro-area** | **Period** | **N** | **BQCV** | **SBV** | **ABPV** | **CBPV** | **DWV** | **KBV** | **IAPV** | **AIV** |
| --- | --- | --- | --- | --- | --- | --- | --- | --- | --- | --- | --- |
| 2009 | N | 1 | 28 | 46.4 | 25.0 | 0.0 | 0.0 | 21.4 | 0.0 | 0.0 | 0.0 |
| 2009 | N | 2 | 28 | 35.7 | 21.4 | 3.6 | 14.3 | 39.3 | 0.0 | 0.0 | 0.0 |
| 2009 | N | 3 | 28 | 60.7 | 0.0 | 7.1 | 7.1 | 60.7 | 0.0 | 0.0 | 0.0 |
| 2009 | N | 4 | 15 | 80.0 | 0.0 | 6.7 | 0.0 | 73.3 | 0.0 | 0.0 | 0.0 |
| 2009 | C | 1 | 43 | 34.9 | 16.3 | 0.0 | 0.0 | 30.2 | 0.0 | 0.0 | 0.0 |
| 2009 | C | 2 | 36 | 50.0 | 13.9 | 5.6 | 8.3 | 52.8 | 0.0 | 0.0 | 0.0 |
| 2009 | C | 3 | 34 | 61.8 | 2.9 | 0.0 | 0.0 | 85.3 | 2.9 | 0.0 | 0.0 |
| 2009 | C | 4 | 25 | 72.0 | 0.0 | 8.0 | 0.0 | 92.0 | 0.0 | 0.0 | 0.0 |
| 2009 | S | 1 | 35 | 37.1 | 20.0 | 5.7 | 14.3 | 42.9 | 0.0 | 0.0 | 0.0 |
| 2009 | S | 2 | 37 | 81.1 | 54.1 | 21.6 | 8.1 | 67.6 | 0.0 | 0.0 | 0.0 |
| 2009 | S | 3 | 37 | 56.8 | 2.7 | 21.6 | 0.0 | 73.0 | 0.0 | 0.0 | 0.0 |
| 2009 | S | 4 | 24 | 83.3 | 0.0 | 25.0 | 0.0 | 91.7 | 0.0 | 0.0 | 0.0 |
| 2010 | N | 1 | 33 | 97.0 | 27.3 | 0.0 | 6.1 | 33.3 | 0.0 | 0.0 | 0.0 |
| 2010 | N | 2 | 32 | 90.6 | 71.9 | 12.5 | 6.3 | 46.9 | 0.0 | 0.0 | 0.0 |
| 2010 | N | 3 | 32 | 96.9 | 50.0 | 15.6 | 12.5 | 75.0 | 12.5 | 0.0 | 0.0 |
| 2010 | N | 4 | 15 | 86.7 | 33.3 | 33.3 | 26.7 | 86.7 | 6.7 | 0.0 | 0.0 |
| 2010 | C | 1 | 35 | 91.4 | 22.9 | 8.6 | 22.9 | 68.6 | 0.0 | 0.0 | 0.0 |
| 2010 | C | 2 | 27 | 88.9 | 51.9 | 11.1 | 18.5 | 77.8 | 0.0 | 0.0 | 0.0 |
| 2010 | C | 3 | 32 | 93.8 | 81.3 | 18.8 | 3.1 | 90.6 | 0.0 | 3.1 | 0.0 |
| 2010 | C | 4 | 14 | 85.7 | 28.6 | 21.4 | 7.1 | 78.6 | 0.0 | 7.1 | 0.0 |
| 2010 | S | 1 | 43 | 97.7 | 65.1 | 44.2 | 9.3 | 81.4 | 0.0 | 0.0 | 0.0 |
| 2010 | S | 2 | 44 | 95.5 | 84.1 | 47.7 | 4.5 | 84.1 | 0.0 | 2.3 | 0.0 |
| 2010 | S | 3 | 45 | 91.1 | 73.3 | 51.1 | 6.7 | 88.9 | 0.0 | 0.0 | 0.0 |
| 2010 | S | 4 | 27 | 92.6 | 55.6 | 55.6 | 7.4 | 92.6 | 0.0 | 0.0 | 0.0 |

N=Northern Italy, C=Central Italy; S=Southern Italy
