# Supplementary material for: The Status of Honey Bee Health in Italy: Results from the Nationwide Bee Monitoring Network
Source: PLoS One. 2016 May 16;11(5):e0155411. doi: 10.1371/journal.pone.0155411 (PMC4868308; doi:10.1371/journal.pone.0155411)
Supplement: S7 Table — (DOCX) [file pone.0155411.s008.docx]

**S7 Table. Percentage of proteins in bee-bread classified per year, macro area and period.**

| **Year** | **Macro area** | **Period** | **N** | **Mean** | **SD** | **SE** |
| --- | --- | --- | --- | --- | --- | --- |
| 2009 | N | 1 | 26 | 17.81 | 2.92 | 0.57 |
| 2009 | N | 2 | 22 | 20.49 | 3.03 | 0.65 |
| 2009 | N | 3 | 19 | 17.98 | 4.25 | 0.98 |
| 2009 | N | 4 | 9 | 18.38 | 2.55 | 0.85 |
| 2009 | C | 1 | 19 | 19.47 | 2.95 | 0.68 |
| 2009 | C | 2 | 23 | 21.63 | 2.94 | 0.61 |
| 2009 | C | 3 | 25 | 19.49 | 3.09 | 0.62 |
| 2009 | C | 4 | 18 | 20.18 | 2.33 | 0.55 |
| 2009 | S | 1 | 31 | 22.04 | 5.54 | 0.99 |
| 2009 | S | 2 | 32 | 21.58 | 4.44 | 0.78 |
| 2009 | S | 3 | 26 | 19.01 | 3.76 | 0.74 |
| 2009 | S | 4 | 15 | 21.39 | 2.48 | 0.64 |
| 2010 | N | 1 | 25 | 19.41 | 4.01 | 0.80 |
| 2010 | N | 2 | 26 | 20.14 | 3.00 | 0.59 |
| 2010 | N | 3 | 24 | 18.95 | 4.02 | 0.82 |
| 2010 | N | 4 | 12 | 19.77 | 2.62 | 0.76 |
| 2010 | C | 1 | 23 | 20.29 | 3.71 | 0.77 |
| 2010 | C | 2 | 20 | 20.45 | 3.00 | 0.67 |
| 2010 | C | 3 | 21 | 19.68 | 3.28 | 0.72 |
| 2010 | C | 4 | 12 | 19.70 | 3.72 | 1.07 |
| 2010 | S | 1 | 23 | 23.58 | 3.16 | 0.66 |
| 2010 | S | 2 | 23 | 22.38 | 3.38 | 0.71 |
| 2010 | S | 3 | 20 | 20.34 | 2.34 | 0.52 |
| 2010 | S | 4 | 19 | 19.70 | 3.79 | 0.87 |

N=Northern Italy, C=Central Italy; S=Southern Italy
