# Supplementary material for: The Status of Honey Bee Health in Italy: Results from the Nationwide Bee Monitoring Network
Source: PLoS One. 2016 May 16;11(5):e0155411. doi: 10.1371/journal.pone.0155411 (PMC4868308; doi:10.1371/journal.pone.0155411)
Supplement: S9 Table — (DOCX) [file pone.0155411.s010.docx]

**S9 Table. Number of positive sample (N) for each compound and percentage of pesticide detection (%) in honey bees, beeswax and bee-bread samples from Italian bee colonies in 2009 and 2010.**

| **#** | **Pesticide** | **Class*** | **2009 (N)** | | | **2009 (%)** | | | **2010 (N)** | | | **2010 (%)** | | |
| --- | --- | --- | --- | --- | --- | --- | --- | --- | --- | --- | --- | --- | --- | --- |
|  |  |  | **Honey bees** | **Beeswax** | **Bee-bread** | **Honey bees** | **Beeswax** | **Bee-bread** | **Honey bees** | **Beeswax** | **Bee-bread** | **Honey bees** | **Beeswax** | **Bee-bread** |
| 1 | Acrinathrine | PYR | 1 | 12 | 6 | 1.5 | 6.0 | 10.0 | 1.0 | 18.0 | 6.0 | 3.7 | 8.6 | 5.1 |
| 2 | Benalaxyl | FUNG | 0 | 0 | 1 | 0.0 | 0.0 | 1.7 | 0.0 | 0.0 | 0.0 | 0.0 | 0.0 | 0.0 |
| 3 | Bitertanol | FUNG | 6 | 1 | 0 | 8.8 | 0.5 | 0.0 | 0.0 | 0.0 | 0.0 | 0.0 | 0.0 | 0.0 |
| 4 | Chlorfenvinphos | OP | 1 | 36 | 2 | 1.5 | 18.1 | 3.3 | 1.0 | 20.0 | 6.0 | 3.7 | 9.5 | 5.1 |
| 5 | Cyprodinil | FUNG | 0 | 3 | 2 | 0.0 | 1.5 | 3.3 | 0.0 | 2.0 | 0.0 | 0.0 | 1.0 | 0.0 |
| 6 | Chlorpyrifos ethil | OP | 2 | 0 | 0 | 2.9 | 0.0 | 0.0 | 0.0 | 0.0 | 0.0 | 0.0 | 0.0 | 0.0 |
| 7 | Clothianidin | NEO | 3 | 0 | 0 | 4.4 | 0.0 | 0.0 | 1.0 | 0.0 | 8.0 | 3.7 | 0.0 | 6.8 |
| 8 | Coumaphos | OP | 11 | 62 | 17 | 16.2 | 31.2 | 28.3 | 2.0 | 59.0 | 17.0 | 7.4 | 28.1 | 14.4 |
| 9 | Dimethoate | OP | 0 | 0 | 0 | 0.0 | 0.0 | 0.0 | 0.0 | 0.0 | 9.0 | 0.0 | 0.0 | 7.6 |
| 10 | Dimethomorph | FUNG | 1 | 0 | 0 | 1.5 | 0.0 | 0.0 | 0.0 | 0.0 | 1.0 | 0.0 | 0.0 | 0.8 |
| 11 | Dithianon | FUNG | 0 | 0 | 0 | 0.0 | 0.0 | 0.0 | 2.0 | 0.0 | 0.0 | 7.4 | 0.0 | 0.0 |
| 12 | Fenamidone | FUNG | 0 | 0 | 0 | 0.0 | 0.0 | 0.0 | 0.0 | 0.0 | 1.0 | 0.0 | 0.0 | 0.8 |
| 13 | Fenbuconazole | FUNG | 0 | 0 | 0 | 0.0 | 0.0 | 0.0 | 1.0 | 0.0 | 0.0 | 3.7 | 0.0 | 0.0 |
| 14 | Fenpyroximate | ACAR | 0 | 0 | 0 | 0.0 | 0.0 | 0.0 | 0.0 | 0.0 | 1.0 | 0.0 | 0.0 | 0.8 |
| 15 | Fipronil | PHE | 0 | 0 | 0 | 0.0 | 0.0 | 0.0 | 0.0 | 1.0 | 0.0 | 0.0 | 0.5 | 0.0 |
| 16 | Fludioxonil | FUNG | 2 | 0 | 3 | 2.9 | 0.0 | 5.0 | 3.0 | 0.0 | 3.0 | 11.1 | 0.0 | 2.5 |
| 17 | Flumetrina | PYR | 1 | 5 | 2 | 1.5 | 2.5 | 3.3 | 4.0 | 14.0 | 5.0 | 14.8 | 6.7 | 4.2 |
| 18 | Fluvalinate | PYR | 10 | 59 | 9 | 14.7 | 29.6 | 15.0 | 5.0 | 83.0 | 32.0 | 18.5 | 39.5 | 27.1 |
| 19 | Imidacloprid | NEO | 7 | 7 | 1 | 10.3 | 3.5 | 1.7 | 1.0 | 0.0 | 2.0 | 3.7 | 0.0 | 1.7 |
| 20 | Kresomix methyl | FUNG | 1 | 0 | 1 | 1.5 | 0.0 | 1.7 | 0.0 | 0.0 | 1.0 | 0.0 | 0.0 | 0.8 |
| 21 | Metalaxil | FUNG | 0 | 1 | 1 | 0.0 | 0.5 | 1.7 | 0.0 | 4.0 | 2.0 | 0.0 | 1.9 | 1.7 |
| 22 | Metamitron | HERB | 0 | 2 | 1 | 0.0 | 1.0 | 1.7 | 0.0 | 0.0 | 0.0 | 0.0 | 0.0 | 0.0 |
| 23 | Methomyl | CARB | 3 | 0 | 0 | 4.4 | 0.0 | 0.0 | 0.0 | 0.0 | 0.0 | 0.0 | 0.0 | 0.0 |
| 24 | Oxamyl | CARB | 0 | 0 | 0 | 0.0 | 0.0 | 0.0 | 0.0 | 0.0 | 2.0 | 0.0 | 0.0 | 1.7 |
| 25 | Piperonyl butoxide | SYN | 0 | 1 | 0 | 0.0 | 0.5 | 0.0 | 0.0 | 0.0 | 0.0 | 0.0 | 0.0 | 0.0 |
| 26 | Pirimicarb | CARB | 0 | 3 | 1 | 0.0 | 1.5 | 1.7 | 1.0 | 4.0 | 1.0 | 3.7 | 1.9 | 0.8 |
| 27 | Propamocarb | FUNG | 0 | 0 | 10 | 0.0 | 0.0 | 16.7 | 2.0 | 1.0 | 12.0 | 7.4 | 0.5 | 10.2 |
| 28 | Pyrimethanil | FUNG | 0 | 0 | 1 | 0.0 | 0.0 | 1.7 | 0.0 | 4.0 | 2.0 | 0.0 | 1.9 | 1.7 |
| 29 | Rotenone | NP | 17 | 6 | 2 | 25.0 | 3.0 | 3.3 | 0.0 | 0.0 | 3.0 | 0.0 | 0.0 | 2.5 |
| 30 | Tebuconazole | FUNG | 0 | 0 | 0 | 0.0 | 0.0 | 0.0 | 2.0 | 0.0 | 1.0 | 7.4 | 0.0 | 0.8 |
| 31 | Teflubenzuron | IGR | 1 | 0 | 0 | 1.5 | 0.0 | 0.0 | 1.0 | 0.0 | 0.0 | 3.7 | 0.0 | 0.0 |
| 32 | Thiacloprid | NEO | 1 | 0 | 0 | 1.5 | 0.0 | 0.0 | 0.0 | 0.0 | 0.0 | 0.0 | 0.0 | 0.0 |
| 33 | Thiametoxam | NEO | 0 | 1 | 0 | 0.0 | 0.5 | 0.0 | 0.0 | 0.0 | 2.0 | 0.0 | 0.0 | 1.7 |
| 34 | Thiophanate methyl | FUNG | 0 | 0 | 0 | 0.0 | 0.0 | 0.0 | 0.0 | 0.0 | 1.0 | 0.0 | 0.0 | 0.8 |
|  | ***N of samples*** | | ***335*** | ***376*** | ***206*** |  |  |  | ***401*** | ***481*** | ***374*** |  |  |  |
|  | ***N different pesticides*** |  | ***16*** | ***14*** | ***16*** |  |  |  | ***14*** | ***11*** | ***22*** |  |  |  |

*Class: ACAR=acaricide, CAR=carbamate, FUNG=fungicide, HERB=herbicide, IGR= Insect Growth Regulator, NEO=neonicotinoid, NP= natural product, OP=organophosphate, PHE=phenylpyrazole, PYR=pyrethroids, SYN= synergist
