# Supplementary material for: The Status of Honey Bee Health in Italy: Results from the Nationwide Bee Monitoring Network
Source: PLoS One. 2016 May 16;11(5):e0155411. doi: 10.1371/journal.pone.0155411 (PMC4868308; doi:10.1371/journal.pone.0155411)
Supplement: S10 Table — Association between pathogens in sampling periods. (DOCX) [file pone.0155411.s011.docx]

**S10 Table. Year 2009. Association between pathogens in sampling periods.**

|  |  | |  |  |  |  |  |  |  |  |  |  |  |  |
| --- | --- | --- | --- | --- | --- | --- | --- | --- | --- | --- | --- | --- | --- | --- |
|  |  | |  | **1st period (n= 81)** | |  | **2nd period (n= 88)** | |  | **3rd period (n= 84)** | |  | **4th period (n= 51)** | |
|  | | **Pathogen pair** | | **phi** | **P-value** |  | **phi** | **P-value** |  | **phi** | **P-value** |  | **phi** | **P-value** |
| ABPV |  | | CBPV | 0.491 | <0.001* |  | -0.121 | 0.262 |  | -0.054 | 0.625 |  | - | - |
| ABPV |  | | DWV | 0.167 | 0.135 |  | 0.319 | 0.002* |  | 0.206 | 0.060 |  | 0.185 | 0.195 |
| ABPV |  | | SBV | -0.064 | 0.570 |  | 0.435 | <0.001* |  | -0.038 | 0.731 |  | - | - |
| ABPV |  | | BQCV | 0.125 | 0.266 |  | 0.298 | 0.005* |  | -0.028 | 0.800 |  | 0.229 | 0.107 |
| ABPV |  | | KBV | - | - |  | - | - |  | -0.038 | 0.731 |  | - | - |
| ABPV |  | | IAPV | - | - |  | - | - |  | - | - |  | - | - |
| ABPV |  | | NC | -0.154 | 0.170 |  | -0.045 | 0.680 |  | 0.192 | 0.079 |  | 0.079 | 0.583 |
| CBPV |  | | DWV | 0.218 | 0.051 |  | 0.075 | 0.489 |  | 0.093 | 0.400 |  | - | - |
| CBPV |  | | SBV | 0.002 | 0.988 |  | 0.083 | 0.445 |  | -0.017 | 0.877 |  | - | - |
| CBPV |  | | BQCV | 0.140 | 0.212 |  | 0.128 | 0.234 |  | 0.129 | 0.243 |  | - | - |
| CBPV |  | | KBV | - | - |  | - | - |  | -0.017 | 0.877 |  | - | - |
| CBPV |  | | IAPV | - | - |  | - | - |  | - | - |  | - | - |
| CBPV |  | | NC | -0.074 | 0.512 |  | 0.164 | 0.128 |  | 0.000 | 1.000 |  | - | - |
| DWV |  | | SBV | 0.113 | 0.314 |  | 0.382 | <0.001* |  | 0.065 | 0.555 |  | - | - |
| DWV |  | | BQCV | 0.371 | <0.001* |  | 0.421 | <0.001* |  | 0.171 | 0.120 |  | 0.377 | 0.006 |
| DWV |  | | KBV | - | - |  | - | - |  | 0.065 | 0.555 |  | - | - |
| DWV |  | | IAPV | - | - |  | - | - |  | - | - |  | - | - |
| DWV |  | | NC | -0.133 | 0.238 |  | -0.159 | 0.139 |  | 0.108 | 0.327 |  | 0.034 | 0.815 |
| SBV |  | | BQCV | 0.179 | 0.109 |  | 0.485 | <0.001* |  | 0.091 | 0.413 |  | - | - |
| SBV |  | | KBV | - | - |  | - | - |  | -0.012 | 0.913 |  | - | - |
| SBV |  | | IAPV | - | - |  | - | - |  | - | - |  | - | - |
| SBV |  | | NC | -0.065 | 0.562 |  | -0.159 | 0.139 |  | -0.110 | 0.320 |  | - | - |
| BQCV |  | | KBV | - | - |  | - | - |  | 0.091 | 0.413 |  | - | - |
| BQCV |  | | IAPV | - | - |  | - | - |  | - | - |  | - | - |
| BQCV |  | | NC | -0.081 | 0.471 |  | -0.111 | 0.304 |  | -0.097 | 0.380 |  | 0.169 | 0.236 |
| KBV |  | | IAPV | - | - |  | - | - |  | - | - |  | - | - |
| KBV |  | | NC | - | - |  | - | - |  | -0.110 | 0.320 |  | - | - |
| IAPV |  | | NC | - | - |  | - | - |  | - | - |  | - | - |

The number of samples for each period is shown in parentheses. ABPV = acute bee paralysis virus; CBPV = chronic bee paralysis virus; DWV = deformed wing virus; SBV = sacbrood virus; BQCV = black queen cell virus; KBV = Kashmir bee virus; IAPV = Israeli acute paralysis virus; NC = *Nosema ceranae*; - = No statistic due to the absence of one of the two pathogens. Asterisk indicates a significant comparison after Bonferroni correction for multiple tests.
