# Supplementary material for: The Status of Honey Bee Health in Italy: Results from the Nationwide Bee Monitoring Network
Source: PLoS One. 2016 May 16;11(5):e0155411. doi: 10.1371/journal.pone.0155411 (PMC4868308; doi:10.1371/journal.pone.0155411)
Supplement: S11 Table — Association between pathogens in sampling periods. (DOCX) [file pone.0155411.s012.docx]

**S11 Table. Year 2010. Association between pathogens in sampling periods.**

|  |  | |  | |  |  |  |  |  |  |  |  |  |  |  |  |
| --- | --- | --- | --- | --- | --- | --- | --- | --- | --- | --- | --- | --- | --- | --- | --- | --- |
|  |  | |  | |  | **1st period (n= 82)** | |  | **2nd period (n= 79)** | |  | **3rd period (n= 80)** | |  | **4th period (n= 46)** | |
|  | |  | | **Pathogen pair** | | **phi** | **P-value** |  | **phi** | **P-value** |  | **phi** | **P-value** |  | **phi** | **P-value** |
| ABPV |  | |  | | CBPV | -0.201 | 0.070 |  | -0.054 | 0.634 |  | -0.037 | 0.746 |  | 0.211 | 0.159 |
| ABPV |  | |  | | DWV | 0.348 | 0.001* |  | 0.117 | 0.305 |  | 0.122 | 0.281 |  | 0.259 | 0.082 |
| ABPV |  | |  | | SBV | 0.188 | 0.090 |  | 0.053 | 0.644 |  | 0.135 | 0.234 |  | 0.244 | 0.102 |
| ABPV |  | |  | | BQCV | 0.005 | 0.967 |  | 0.167 | 0.142 |  | 0.069 | 0.544 |  | 0.355 | 0.015 |
| ABPV |  | |  | | KBV | - | - |  | - | - |  | -0.037 | 0.746 |  | -0.125 | 0.408 |
| ABPV |  | |  | | IAPV | - | - |  | -0.073 | 0.525 |  | - | - |  | 0.178 | 0.237 |
| ABPV |  | |  | | NC | -0.106 | 0.343 |  | -0.131 | 0.248 |  | -0.118 | 0.299 |  | 0.004 | 0.977 |
| CBPV |  | |  | | DWV | -0.039 | 0.725 |  | -0.158 | 0.165 |  | 0.092 | 0.419 |  | 0.095 | 0.529 |
| CBPV |  | |  | | SBV | -0.007 | 0.950 |  | -0.066 | 0.563 |  | -0.066 | 0.563 |  | 0.041 | 0.789 |
| CBPV |  | |  | | BQCV | -0.049 | 0.660 |  | -0.234 | 0.038 |  | -0.415 | <0.001* |  | -0.084 | 0.579 |
| CBPV |  | |  | | KBV | - | - |  | - | - |  | -0.053 | 0.643 |  | -0.046 | 0.761 |
| CBPV |  | |  | | IAPV | - | - |  | -0.041 | 0.722 |  | - | - |  | -0.046 | 0.761 |
| CBPV |  | |  | | NC | 0.050 | 0.654 |  | -0.052 | 0.647 |  | -0.048 | 0.672 |  | -0.183 | 0.223 |
| DWV |  | |  | | SBV | 0.160 | 0.150 |  | 0.331 | 0.003* |  | 0.001 | 0.993 |  | 0.115 | 0.447 |
| DWV |  | |  | | BQCV | 0.264 | 0.016 |  | 0.327 | 0.003* |  | -0.103 | 0.363 |  | 0.299 | 0.044 |
| DWV |  | |  | | KBV | - | - |  | - | - |  | -0.075 | 0.509 |  | 0.046 | 0.761 |
| DWV |  | |  | | IAPV | - | - |  | 0.066 | 0.564 |  | - | - |  | 0.046 | 0.761 |
| DWV |  | |  | | NC | 0.154 | 0.168 |  | -0.053 | 0.641 |  | -0.011 | 0.920 |  | 0.008 | 0.960 |
| SBV |  | |  | | BQCV | 0.032 | 0.773 |  | 0.327 | 0.003* |  | 0.020 | 0.859 |  | 0.127 | 0.399 |
| SBV |  | |  | | KBV | - | - |  | - | - |  | -0.185 | 0.101 |  | 0.170 | 0.259 |
| SBV |  | |  | | IAPV | - | - |  | 0.066 | 0.564 |  | - | - |  | -0.131 | 0.386 |
| SBV |  | |  | | NC | 0.060 | 0.590 |  | 0.006 | 0.958 |  | 0.117 | 0.300 |  | -0.022 | 0.886 |
| BQCV |  | |  | | KBV | - | - |  | - | - |  | 0.059 | 0.602 |  | -0.352 | 0.016 |
| BQCV |  | |  | | IAPV | - | - |  | 0.029 | 0.797 |  | - | - |  | 0.063 | 0.677 |
| BQCV |  | |  | | NC | 0.192 | 0.084 |  | 0.003 | 0.981 |  | -0.027 | 0.812 |  | 0.114 | 0.451 |
| KBV |  | |  | | IAPV | - | - |  | - | - |  | - | - |  | -0.022 | 0.883 |
| KBV |  | |  | | NC | - | - |  | - | - |  | -0.048 | 0.672 |  | -0.089 | 0.558 |
| IAPV |  | |  | | NC | - | - |  | -0.093 | 0.413 |  | - | - |  | 0.251 | 0.093 |

The number of samples for each period is shown in parentheses. ABPV = acute bee paralysis virus; CBPV = chronic bee paralysis virus; DWV = deformed wing virus; SBV = sacbrood virus; BQCV = black queen cell virus; KBV = Kashmir bee virus; IAPV = Israeli acute paralysis virus; NC = *Nosema ceranae*; - = No statistic due to the absence of one of the two pathogens. Asterisk indicates a significant comparison after Bonferroni correction for multiple tests.
