# Supplementary material for: The Status of Honey Bee Health in Italy: Results from the Nationwide Bee Monitoring Network
Source: PLoS One. 2016 May 16;11(5):e0155411. doi: 10.1371/journal.pone.0155411 (PMC4868308; doi:10.1371/journal.pone.0155411)
Supplement: S12 Table — (DOCX) [file pone.0155411.s013.docx]

**S12 Table. Percentage of colony losses per each monitored Italian region and year (N = number of apiaries per each region).**

| **Region** | **Annual colony losses (2009)** | | | **Seasonal colony**  **losses**  **(2010)** | | |
| --- | --- | --- | --- | --- | --- | --- |
|  | **N** | **Mean** | **SD** | **N** | **Mean** | **SD** |
| Trentino-Alto Adige | 8 | 7.50 | 14.88 | 5 | 4.0 | 8.9 |
| Veneto | 5 | 32.00 | 16.43 | 7 | 7.1 | 11.1 |
| Emilia Romagna | 10 | 15.00 | 30.64 | 15 | 13.3 | 14.0 |
| Liguria | 5 | 18.00 | 4.47 | 6 | 0.0 | 0.0 |
| Toscana | 5 | 18.00 | 24.90 | 5 | 0.0 | 0.0 |
| Marche | 5 | 24.00 | 18.17 | 5 | 2.2 | 4.9 |
| Umbria | 6 | 26.67 | 28.05 | 8 | 13.8 | 11.9 |
| Abruzzo | 5 | 4.00 | 8.94 | 1 | 10.0 |  |
| Lazio | 5 | 30.00 | 15.81 | 5 | 8.0 | 13.0 |
| Molise | NA | NA | NA | 5 | 0.0 | 0.0 |
| Sardegna | 5 | 16.00 | 13.42 | 4 | 15.0 | 30.0 |
| Puglia | 5 | 34.00 | 23.02 | 5 | 14.0 | 11.4 |
| Campania | 5 | 30.00 | 23.45 | 1 | 20.0 |  |
| Basilicata | 5 | 4.00 | 8.94 | 5 | 8.0 | 17.9 |
| Calabria | 5 | 18.00 | 10.95 | 16 | 2.5 | 6.8 |
| Sicilia | 5 | 20.00 | 10.00 | 4 | 7.5 | 5.0 |

NA = not available
